# Metabolic engineering of *Corynebacterium glutamicum* for fatty alcohol production from glucose and wheat straw hydrolysate

**DOI:** 10.1186/s13068-023-02367-3

**Published:** 2023-07-18

**Authors:** Felix Werner, Lynn S. Schwardmann, Daniel Siebert, Christian Rückert-Reed, Jörn Kalinowski, Marie-Theres Wirth, Katharina Hofer, Ralf Takors, Volker F. Wendisch, Bastian Blombach

**Affiliations:** 1 Microbial Biotechnology, Campus Straubing for Biotechnology and Sustainability, Technical University of Munich, Uferstraße 53, 94315 Straubing, Germany; 2 Genetics of Prokaryotes, Faculty of Biology and CeBiTec, Bielefeld University, Bielefeld, Germany; 3 SynBiofoundry@TUM, Technical University of Munich, Straubing, Germany; 4 Microbial Genomics and Biotechnology, CeBiTec, Bielefeld University, Bielefeld, Germany; 5 Institute of Biochemical Engineering, University of Stuttgart, Stuttgart, Germany

**Keywords:** Fatty alcohols, Fatty acids, l-Glutamate, l-Lysine, NMePhe, DPA, *Corynebacterium glutamicum*, Xylose, Adaptive laboratory evolution, Wheat straw hydrolysate

## Abstract

**Background:**

Fatty acid-derived products such as fatty alcohols (FAL) find growing application in cosmetic products, lubricants, or biofuels. So far, FAL are primarily produced petrochemically or through chemical conversion of bio-based feedstock. Besides the well-known negative environmental impact of using fossil resources, utilization of bio-based first-generation feedstock such as palm oil is known to contribute to the loss of habitat and biodiversity. Thus, the microbial production of industrially relevant chemicals such as FAL from second-generation feedstock is desirable.

**Results:**

To engineer *Corynebacterium glutamicum* for FAL production, we deregulated fatty acid biosynthesis by deleting the transcriptional regulator gene *fasR*, overexpressing a fatty acyl-CoA reductase (FAR) gene of *Marinobacter hydrocarbonoclasticus* VT8 and attenuating the native thioesterase expression by exchange of the ATG to a weaker TTG start codon. *C. glutamicum* ∆*fasR* cg2692_TTG_ (pEKEx2-*maqu2220*) produced in shaking flasks 0.54 ± 0.02 g_FAL_ L^−1^ from 20 g glucose L^−1^ with a product yield of 0.054 ± 0.001 Cmol Cmol^−1^. To enable xylose utilization, we integrated *xylA* encoding the xylose isomerase from *Xanthomonas campestris* and *xylB* encoding the native xylulose kinase into the locus of *actA*. This approach enabled growth on xylose. However, adaptive laboratory evolution (ALE) was required to improve the growth rate threefold to 0.11 ± 0.00 h^−1^. The genome of the evolved strain *C. glutamicum* gX was re-sequenced, and the evolved genetic module was introduced into *C. glutamicum* ∆*fasR* cg2692_TTG_ (pEKEx2-*maqu2220*) which allowed efficient growth and FAL production on wheat straw hydrolysate. FAL biosynthesis was further optimized by overexpression of the *pntAB* genes encoding the membrane-bound transhydrogenase of *E. coli*. The best-performing strain *C. glutamicum* ∆*fasR* cg2692_TTG_ CgLP12::(P_*tac*_-*pntAB*-T_*rrnB*_) gX (pEKEx2-*maqu2220*) produced 2.45 ± 0.09 g_FAL_ L^−1^ with a product yield of 0.054 ± 0.005 Cmol Cmol^−1^ and a volumetric productivity of 0.109 ± 0.005 g_FAL_ L^−1^ h^−1^ in a pulsed fed-batch cultivation using wheat straw hydrolysate.

**Conclusion:**

The combination of targeted metabolic engineering and ALE enabled efficient FAL production in *C. glutamicum* from wheat straw hydrolysate for the first time. Therefore, this study provides useful metabolic engineering principles to tailor this bacterium for other products from this second-generation feedstock.

**Supplementary Information:**

The online version contains supplementary material available at 10.1186/s13068-023-02367-3.

## Introduction

*Corynebacterium glutamicum* is an established workhorse for the large-scale production of several amino acids, such as l-lysine and l-glutamate, in millions of tons per year [83]. This facultative anaerobic Gram-positive bacterium is generally recognized as safe (GRAS), robust, and grows on several sugars, organic acids, and phenolic compounds as single or combined carbon and energy sources [6, 22, 47, 53, 70, 73]. The naturally accessible carbon source spectrum was expanded by the introduction of heterologous pathways [89], among others, to access xylose, which was first attained by heterologous overexpression of xylose isomerase encoding gene *xylA* from *E. coli* in combination with native xylulokinase activity and thereupon improved by co-overexpression of *xylA* and *xylB* genes from different origins [36, 53]. *Corynebacterium glutamicum* shows a comparable high tolerance to inhibitors such as aromatic compounds typically found in lignocellulosic hydrolysates [6, 20, 69, 70]. Therefore, in several studies, this bacterium was utilized for the production of chemicals and fuels such as lactic, succinic, *cis*, *cis*-muconic, itaconic, 5-aminovaleric acid or 1,2-propanediol and isobutanol from non-food biomass hydrolysates [11, 39, 40, 46, 48, 62, 63]. The non-oleaginous *C. glutamicum* lacks essential genes for the β-oxidation of fatty acids [4] and has already been engineered for the production of lipids and fatty acids (FA) [33, 54, 72, 74]. However, the production of fatty alcohols (FAL) and derivates thereof with *C. glutamicum* has not been described so far.

Because of their broad application as detergents, lubricants, and additives in cosmetic products [1, 21], a continuously increasing demand for FAL is expected [51]. Commercially, FAL are either produced petrochemically by the oligomerization and subsequent oxidation of ethylene [38], or by hydrogenation of bio-based fatty acids and fatty acid methyl esters [86]. Besides the required usage of expensive metal catalysts and the high energy demand [58], the use of fossil resources and extensive farming of oil plants in monocultures propagate global warming, deforestation, and subsequently, loss of biodiversity [26, 79]. Thus, microbial production is an alternative approach for the sustainable production of FAL, especially when agricultural waste or side streams are utilized as substrate.

For the biosynthesis of FAL, two well-described pathways are commonly applied: (A) reduction of a free FA or of acyl-ACP/CoA to fatty aldehydes by a carboxylic acid reductase (CAR) or, respectively, by an aldehyde-forming fatty acyl-ACP/CoA reductase (AH-FAR) with a subsequent second reduction to a long-chain alcohol by an alcohol dehydrogenase (ADH) or aldehyde reductase (AHR) [1, 90] or (B) two-step reduction of an activated FA by an alcohol-forming fatty acyl-CoA reductase (FAR) [16, 43] with an aldehyde intermediate that is formed during the four-electron transfer but which is not released from the enzyme [32, 85]. Especially the latter pathway has been extensively exploited to heterologously produce FAL in various organisms such as *Escherichia coli* [43], *Saccharomyces cerevisiae* [18], and *Yarrowia lipolytica* [16] as it solely requires the expression of one gene and does not form free cytotoxic aldehyde intermediates. The commonly used, NADPH-dependent FAR enzymes Maqu_2220 and Maqu_2507 of the marine bacterium *Marinobacter hydrocarbonoclasticus* VT8 were shown to accept both acyl-ACP and acyl-CoA as substrate, with a higher affinity for C16-C18 acyl-CoAs [32, 85]. This renders both reductases promising candidates to be expressed in *C. glutamicum*, as its native FA biosynthesis primarily produces palmityl-, stearyl- and oleoyl-CoA as intermediates [35, 67]. The pathway’s precursor malonyl-CoA is supplied by the carboxylation of acetyl-CoA, catalyzed by the acetyl-CoA carboxylase (ACC) [27]. In contrast to many other prokaryotes, the successive condensation and elongation reactions of FA biosynthesis in *C. glutamicum* are catalyzed by two type I fatty acid synthases (FAS-I) [57]. Thus, in contrast to ACP-bound thioesters found in FAS-II-utilizing microbes, not only substrates but also products of the two multienzymes Fas-IA and Fas-IB are CoA-bound [57]. Hydrolysis of the respective thioesters catalyzed by an acyl-CoA thioesterase (Tes) results in the formation of the free FAs palmitic acid (hexadecenoic acid), stearic acid (octadecanoic acid) and the monounsaturated oleic acid (*cis*-9-octadecenoic acid) [33]. Those FA serve further as precursors for membrane lipid and mycolic acid biosynthesis. In the presence of acyl-CoAs, FA biosynthesis is tightly regulated by FasR. The TetR-type transcriptional regulator inhibits transcription of both FAS-I-encoding genes *fasA* and *fasB*, and of the ACC catalytic subunit-encoding genes *accD1* and *accBC* [34, 52].

**Fig. 1:**
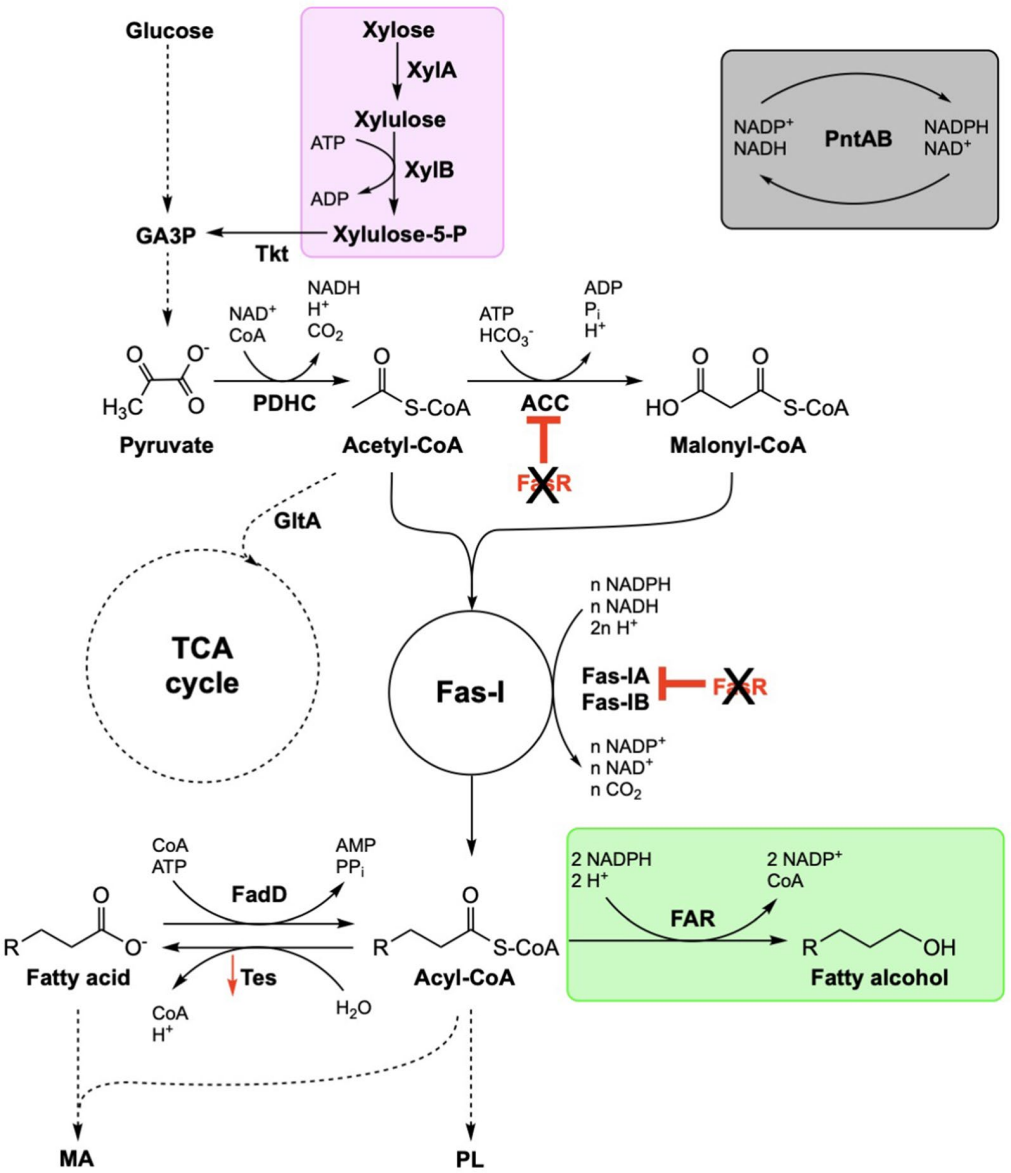
Metabolic pathways for fatty acid biosynthesis and heterologous FAL production in *C. glutamicum*. The heterologous modules for FAL synthesis, xylose utilization, and NADPH regeneration are highlighted in green, purple, and grey, respectively. *ACC* acetyl-CoA carboxylase, *FadD* acyl-CoA synthetase, *FAR* fatty acyl-CoA reductase, *Fas-IA* type I fatty acid synthase A, *Fas-IB* type I fatty acid synthase B, *GltA* citrate synthase, *MA* mycolic acid, *PDHC* pyruvate dehydrogenase complex, *PL* phospholipid, *PntAB* transhydrogenase, *Tkt* transketolase, *XylA* xylose isomerase, *XylB* xylulokinase. The red arrow and the black X represent decreased expression and deletion of the corresponding gene, respectively

To tailor *C. glutamicum* for FAL production, we systematically engineered the FA metabolism and its regulation, optimized the culture conditions, and enabled ALE-supported xylose utilization (Fig. 1). Finally, we validated the performance of the newly constructed strain in a bioreactor setup using wheat straw hydrolysate.

## Results

### Transcriptional deregulation of FA biosynthesis to improve precursor supply for FAL production

**Fig. 2:**
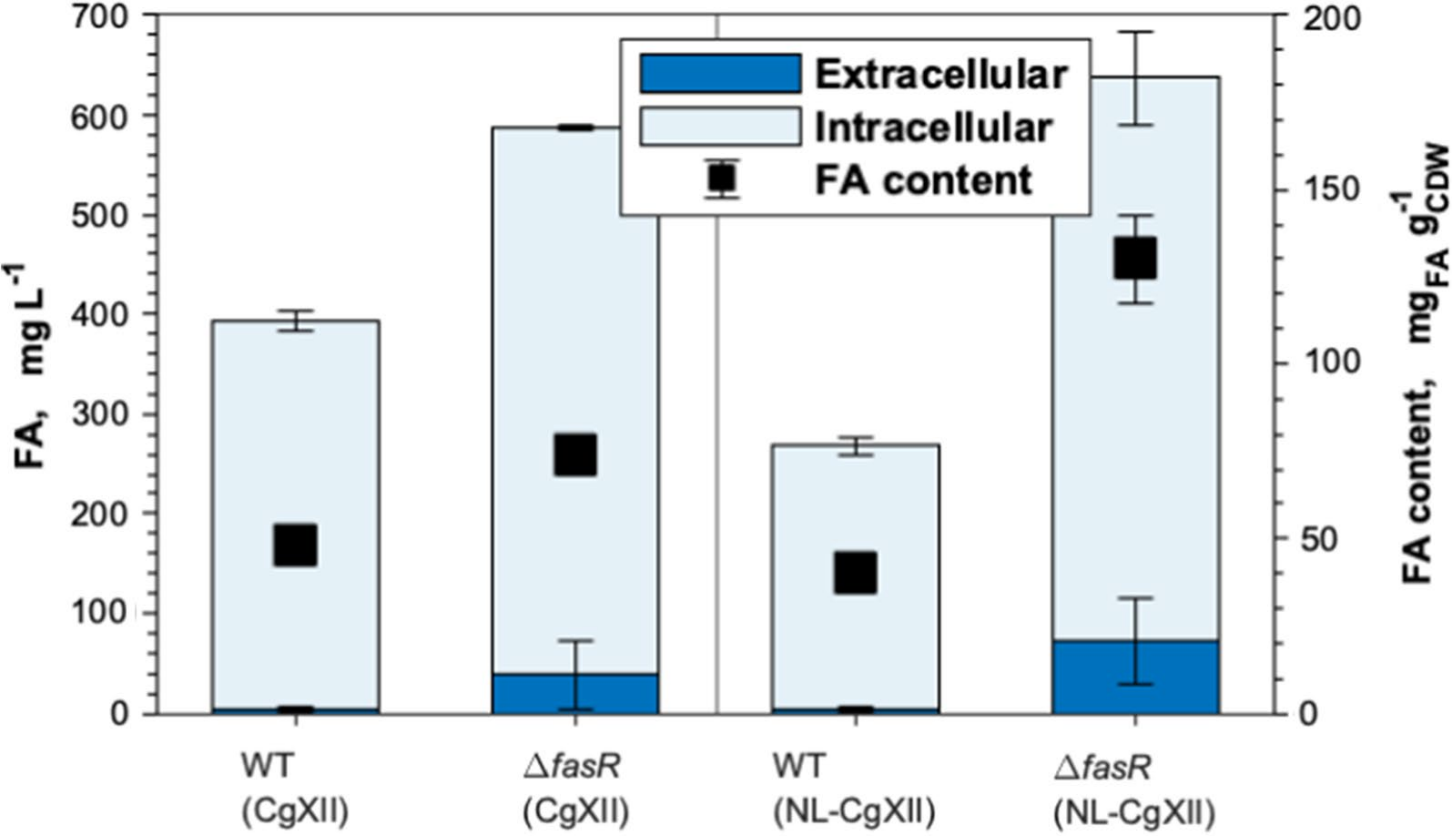
Fatty acid production in CgXII and NL-CgXII medium. Cultivations of *C. glutamicum* WT and the Δ*fasR* mutant were conducted in standard CgXII and in the nitrogen-limiting NL-CgXII medium with 20 g glucose L^−1^. Samples for analysis were taken after 24 h. Data represent means of ≥ 3 biological replicates with standard deviations

As the biosynthetic pathways of FA and FAL synthesis are similar until they diverge at the acyl-CoA node, first, the flux through FA biosynthesis was improved to provide sufficient acyl-CoA for FAL production in *C. glutamicum*. In that pursuit, the transcriptional regulator-encoding gene *fasR* was deleted in *C. glutamicum* WT to derepress the native FA biosynthesis (Fig. 2). The resulting increase of total FA production from 393 ± 13 to 587 ± 37 mg_FA_ L^−1^ was mostly contributed by an increase of intracellular FA (Fig. 2). Upon cultivation in nitrogen-limiting NL-CgXII medium containing 1.45 g urea L^−1^ as the sole nitrogen source, FA efflux was observed only for the Δ*fasR* mutant which secreted 72 ± 42 mg_FA_ L^−1^ into the culture broth. Simultaneously, the FA content of *C. glutamicum* Δ*fasR* increased from 74 ± 3 to 130 ± 12 mg_FA_ g_CDW_^−1^ (Fig. 2; Additional file 1: Figure S1). The relative FA content of the WT remained almost unchanged under both cultivation conditions. Similarly, the distribution of the two quantified products, palmitic and oleic acid, remained almost unchanged for both strains under all tested conditions. Palmitic acid accounted for 48% (w/w) and 47% (w/w) of the FA formed by the WT cultivated in CGXII or NL-CgXII medium, respectively. Likewise, the palmitic acid contents were 53% (w/w) and 49% (w/w) of the quantified FA formed by the Δ*fasR* mutant cultivated in CgXII medium or under nitrogen-limiting conditions, respectively. The remaining fractions represent the respective relative oleic acid contents. Consequently, the nitrogen-limited NL-CgXII medium was used for all subsequent cultivations.

### Enabling FAL production by expression of heterologous FAR

**Fig. 3:**
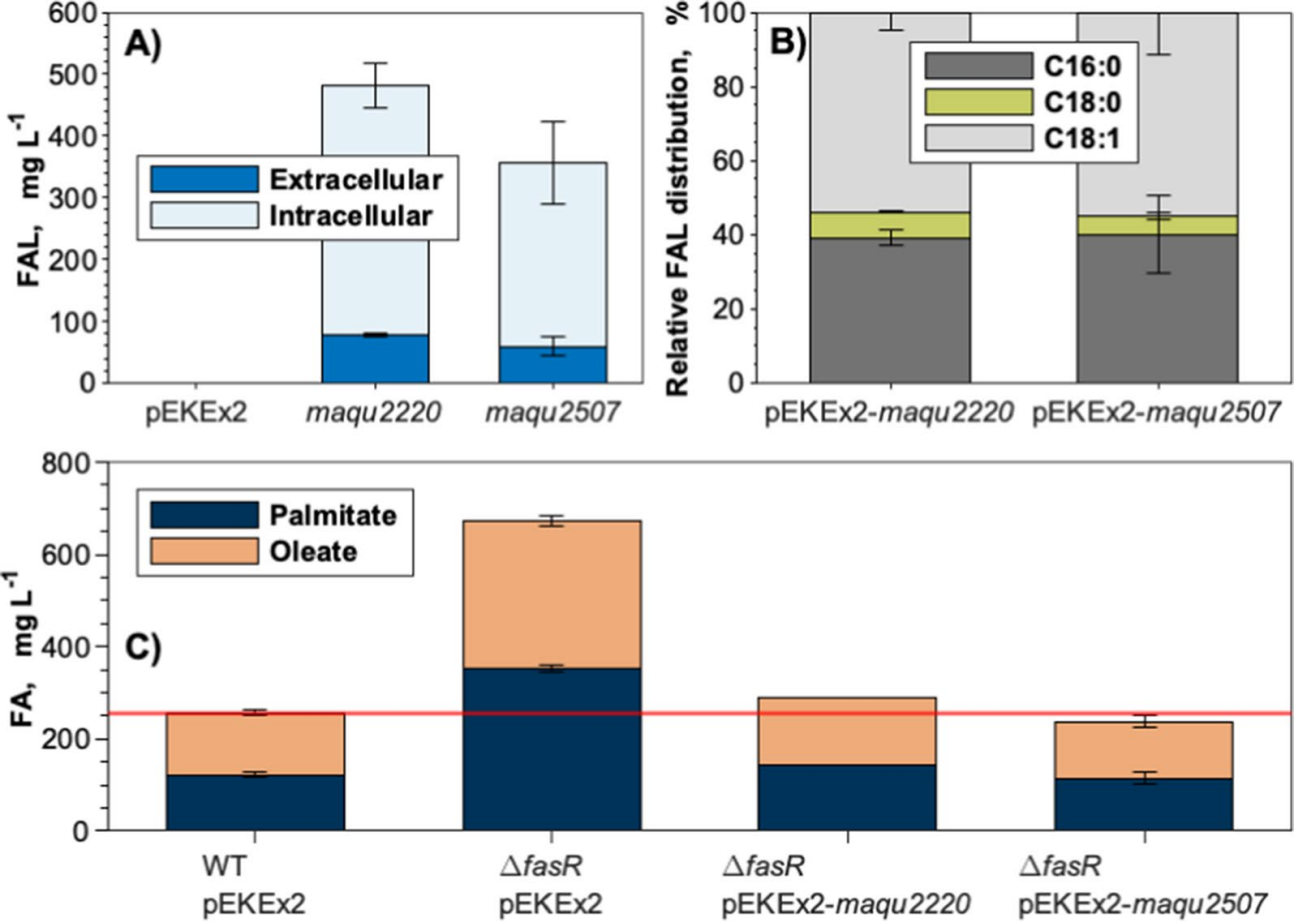
FAL production in *C. glutamicum* Δ*fasR* (pEKEx2-*maqu2220*) and *C. glutamicum* Δ*fasR* (pEKEx2-*maqu2507*) and its effect on FA synthesis. **A** Extra- and intracellular FAL concentrations obtained by Δ*fasR* mutants harboring pEKEx2-*maqu2220* or pEKEx2-*maqu2507*; **B** distribution of 1-hexadecanol (C16:0), octadecanol (C18:0) and oleyl alcohol (C18:1) of the total FAL; **C** total FA produced by the FAR-expressing strains in comparison to WT and Δ*fasR* empty plasmid controls. The red horizontal line indicates the FA level of the WT. Samples for FAL and FA analysis were taken after 24 h. Cultivations were conducted in NL-CGXII containing 20 g glucose L^−1^. Data represent means of ≥ 3 biological replicates with standard deviations

Since *C. glutamicum* does not naturally produce FAL, two fatty acyl-CoA reductases (FAR) of *Marinobacter hydrocarbonoclasticus* VT8 were tested for their suitability to enable FAL production in *C. glutamicum*. For that purpose, the genes encoding the reductases Maqu_2220 and Maqu_2507 were cloned into the plasmid pEKEx2 and expressed in the FA-producing Δ*fasR* mutant. The resulting strains *C. glutamicum* Δ*fasR* (pEKEx2-*maqu2220*) and *C. glutamicum* Δ*fasR* (pEKEx2-*maqu2507*) produced 482 ± 33 mg_FAL_ L^−1^ and 356 ± 82 mg_FAL_ L^−1^ of which 78 ± 3 mg_FAL_ L^−1^ and 59 ± 15 mg_FAL_ L^−1^ were measured extracellularly, respectively (Fig. 3A). The majority of produced FAL were 1-hexadecanol and oleyl alcohol. Less than 10% of the total amount of formed FAL was attributed to 1-octadecanol (Fig. 3B). As expected, the FA production decreased in FAL-producing strains, reaching FA concentrations comparable to the WT (Fig. 3C).

In order to construct a plasmid-free FAL producer, the gene encoding the better-performing reductase Maqu_2220 was integrated into the stable landing pad CgLP11 [40] under the control of P_*tac*_ and terminated by T_*rrnB*_. However, the newly constructed strain *C. glutamicum* Δ*fasR* CgLP11::(P_*tac*_-*maqu_2220*-T_*rrnB*_) produced 94% less FAL than the plasmid-harboring control with 31 ± 2 mg_FAL_ L^−1^ of which 8 ± 2 mg_FAL_ L^−1^ measured extracellularly (Additional file 1: Figure S2). This is commensurate with a reduced gene dose since pEKEx2 has a plasmid copy number between 10 and 30 [22]. Due to the indicated correlation between gene number and FAL production, alternative strategies were pursued to improve FAL production.

### Attenuation of thioesterase expression

**Fig. 4:**
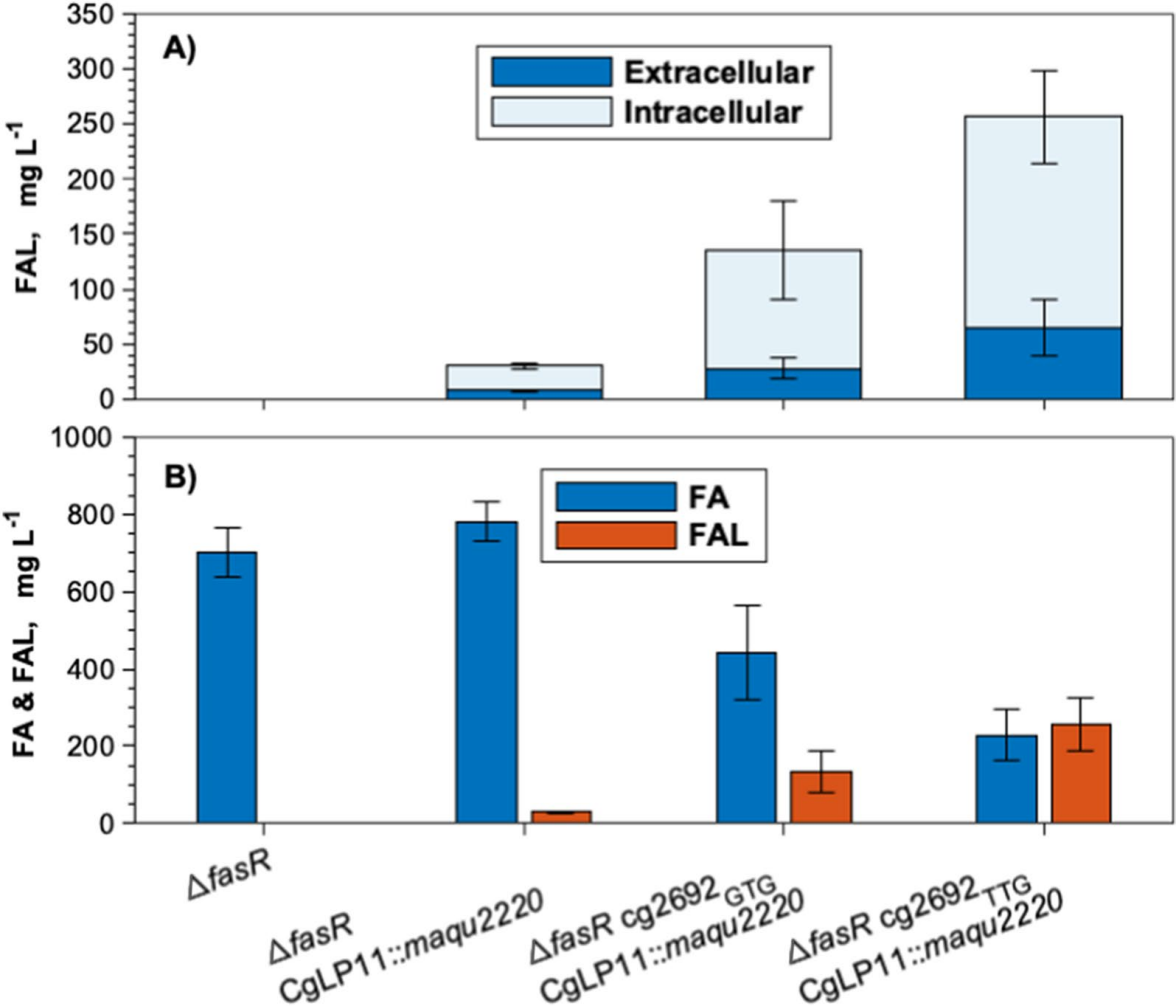
FAL production of plasmid-free *C. glutamicum* Δ*fasR* CgLP11::(P_*tac*_-*maqu_2220*-T_*rrnB*_) mutants differing in the start codon of the thioesterase-encoding cg2692. Cultivations were conducted in NL-CgXII medium containing 20 g glucose L^−1^. **A** Extra- and intracellular FAL concentrations; **B** total FA and FAL concentrations. Samples for FA and FAL analysis were taken after 48 h. Data represent means of 3 biological replicates with standard deviations

Thioesterase-catalyzed FA-forming reactions provide a metabolic sink for acyl-CoA and therefore compete with the FAR-catalyzed reduction towards the formation of FAL. Compared to *E. coli*, *C. glutamicum* possesses a high thioesterase activity [74], and thus, it seemed crucial to attenuate thioesterase expression in order to favor the FAL-forming reduction. Since deleting the native thioesterase-encoding gene cg2692 was shown to severely impact the growth of *C. glutamicum* [33], translational attenuation was chosen as a strategy to redirect the carbon flux. For that purpose, the native start codon ATG of cg2692 was exchanged for the less-preferred GTG and TTG codons in *C. glutamicum* Δ*fasR* CgLP11::(P_*tac*_-*maqu_2220*-T_*rrnB*_). FAL production of the resulting strains *C. glutamicum* Δ*fasR* cg2692_GTG_ CgLP11::(P_*tac*_-*maqu_2220*-T_*rrnB*_) and *C. glutamicum* Δ*fasR* cg2692_TTG_ CgLP11::(P_*tac*_-*maqu_2220*-T_*rrnB*_) was analyzed and compared to the parental strain (Fig. 4A). Upon exchange of the start codon to either GTG or TTG, FAL production increased by 350% and 750%, respectively, accompanied by a substantial reduction in FA production. The highest FAL titer was obtained by *C. glutamicum* Δ*fasR* cg2692_TTG_ CgLP11::(P_*tac*_-*maqu_2220*-T_*rrnB*_) with 256 ± 67 mg_FAL_ L^−1^ which displayed the lowest total FA concentration of 229 ± 64 mg_FA_ L^−1^ (Fig. 4B). The biomass concentrations measured after 48 h remained unaffected by the start codon mutations (data not shown).

**Fig. 5:**
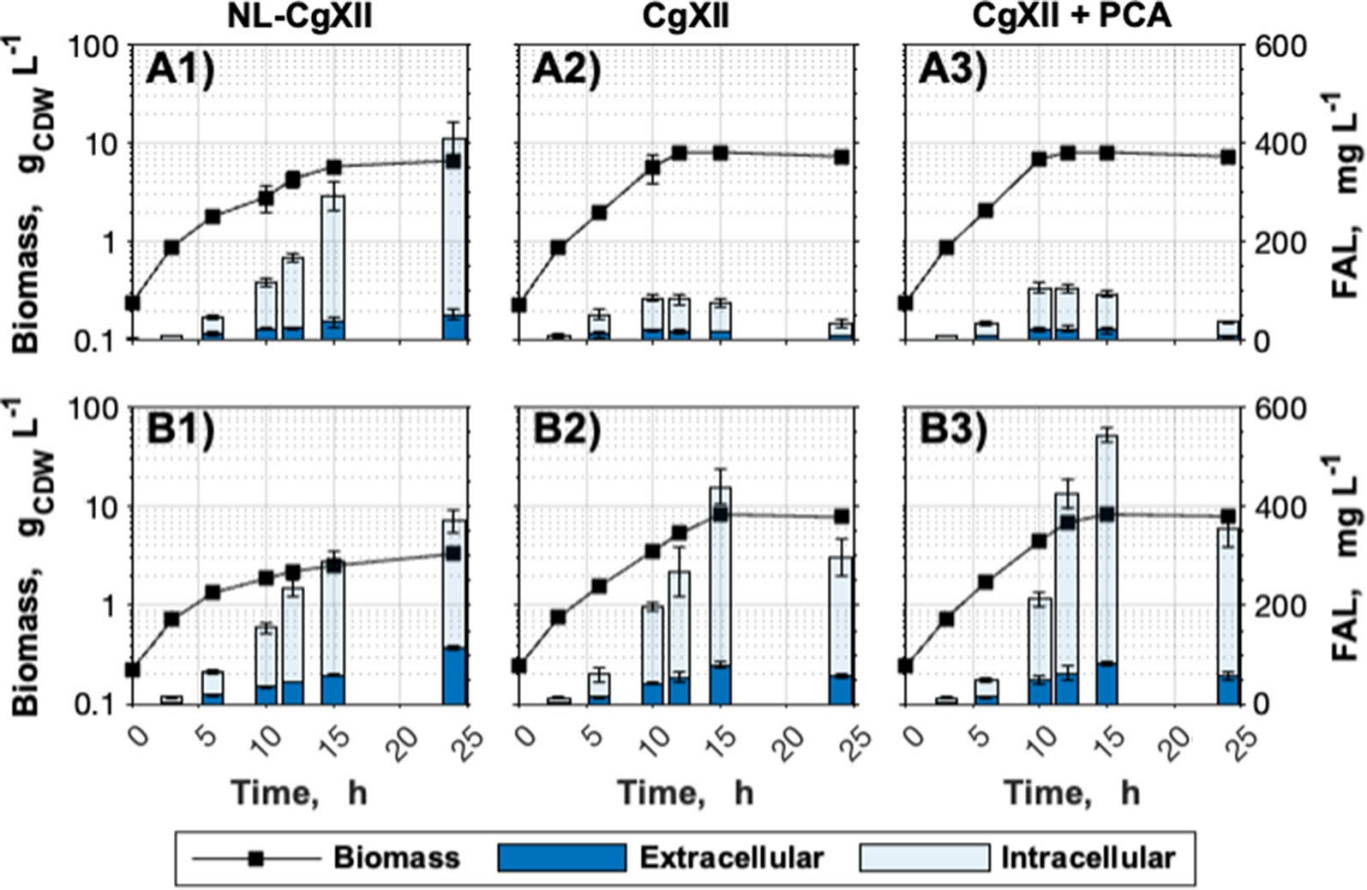
Optimization of the medium composition for the efficient production of FAL. The two best-performing strains **A** *C. glutamicum* Δ*fasR* (pEKEx2-*maqu2220*) and **B** *C. glutamicum* Δ*fasR* cg2692_TTG_ (pEKEx2-*maqu2220*) were cultivated in (1) NL-CgXII, (2) CgXII or (3) CgXII medium supplemented with 195 µM PCA. All media contained 20 g glucose L^−1^ as carbon source. Data represent means of 3 biological replicates with standard deviations

Since a positive correlation between FAL production and gene copy number of *maqu_2220* was assumed, we transformed *C. glutamicum* Δ*fasR* cg2692_TTG_ with the plasmid pEKEx2-*maqu2220*. Compared to *C. glutamicum* Δ*fasR* (pEKEx2-*maqu2220*), *C. glutamicum* Δ*fasR* cg2692_TTG_ (pEKEx2-*maqu2220*) showed a 10% reduction of the total FAL production. However, the extracellular FAL concentration increased by about 120% to 114 ± 4 mg_FA_ L^−1^ in NL-CgXII medium (Fig. 5A1, B1). In contrast to the earlier described FA synthesis (Additional file 1: Figure S1), FAL was formed primarily in a growth-coupled manner. Additionally, we observed that glucose consumption of *C. glutamicum* Δ*fasR* cg2692_TTG_ (pEKEx2-*maqu2220*) ceased after 24 h (data not shown), indicating sub-optimal cultivation conditions.

To test whether FAL production could be improved by solely adjusting the cultivation conditions, *C. glutamicum* Δ*fasR* (pEKEx2-*maqu2220*) and *C. glutamicum* Δ*fasR* cg2692_TTG_ (pEKEx2-*maqu2220*) were cultivated in standard CgXII medium with and without the iron chelator protocatechuic acid (PCA). The compound is often supplemented to *C. glutamicum* minimal media due to its growth-promoting effect [42, 50]. Interestingly, while FAL production by the former strain plummeted under both conditions, growth-coupled FAL production by the latter strain was strongly improved in CgXII medium and in CgXII supplemented with PCA compared to nitrogen-limited medium (Fig. 5B1-3). *C. glutamicum* Δ*fasR* cg2692_TTG_ (pEKEx2-*maqu2220*) produced within 15 h 544 ± 20 mg_FAL_ L^−1^ in CgXII medium supplemented with PCA which corresponds to a 33% increase of the total FAL titer, a 35% higher product yield (i.e., 0.054 ± 0.001 Cmol Cmol^−1^) and a 112% improved volumetric productivity compared to *C. glutamicum* Δ*fasR* (pEKEx2-*maqu2220*) cultivated under nitrogen-limiting conditions (Fig. 5; A2-3, B2-3). Thus, all subsequent cultivations were conducted using the optimized cultivation conditions and the best-performing FAL producer *C. glutamicum* Δ*fasR* cg2692_TTG_ (pEKEx2-*maqu2220*).

### Engineering *C. glutamicum* for xylose utilization

We aimed to establish FAL production from wheat straw hydrolysate, which contained significant amounts of xylose besides glucose and acetate. Since *C. glutamicum* is unable to utilize this C5 sugar, a synthetic operon consisting of *xylA* encoding the xylose isomerase from *Xanthomonas campestris* SCC1758 and *xylB* encoding the xylulose kinase from *C. glutamicum* ATCC 13032 was integrated into the locus of *actA* (cg2840, also known as *cat*) replacing the *actA* open reading frame. The resulting strain *C. glutamicum* Δ*actA*::*xylAB* showed growth on 4% xylose as the sole carbon source. However, the strain grew with a low µ_max_ of 0.03 ± 0.01 h^−1^ and reached a maximal biomass concentration of only 1.7 ± 0.1 g L^−1^ (Additional file 1: Figure S4A). To improve the growth properties of *C. glutamicum* Δ*actA*::*xylAB* on xylose, we applied adaptive laboratory evolution (ALE). Therefore, we cultivated this strain in serial batches using CgXII medium supplemented with 40 g xylose L^−1^ as the sole carbon source with the aim to obtain an isolate with doubled µ_max_. After five serial transfers, a mutant that grew faster and to higher biomass concentrations than the parental strain, was isolated and named *C. glutamicum* gX (Additional file 1: Figure S3). The evolved strain *C. glutamicum* gX showed growth on xylose with more than threefold higher µ_max_ (0.11 ± 0.00 h^−1^) than the parental strain *C. glutamicum* Δ*actA*::*xylAB* and reached a maximal biomass concentration of 5.6 ± 0.3 g L^−1^ (Additional file 1: Figure S4A).

To identify candidate mutations that caused this growth acceleration, the whole genome of strain *C. glutamicum* gX was sequenced. Surprisingly, the only genetic difference to the parental strain *C. glutamicum* Δ*actA*::*xylAB* was a duplication of 53 bp. This rearrangement duplicated a region spanning 21 bp of the UTR sequence and the first 32 bp of the *xylA* open reading frame and resulted in two occurrences of a predicted RBS (AGGAG) at 6 and 59 bp upstream of the start codon of *xylA* (Additional file 1: Figure S4B).

**Fig. 6:**
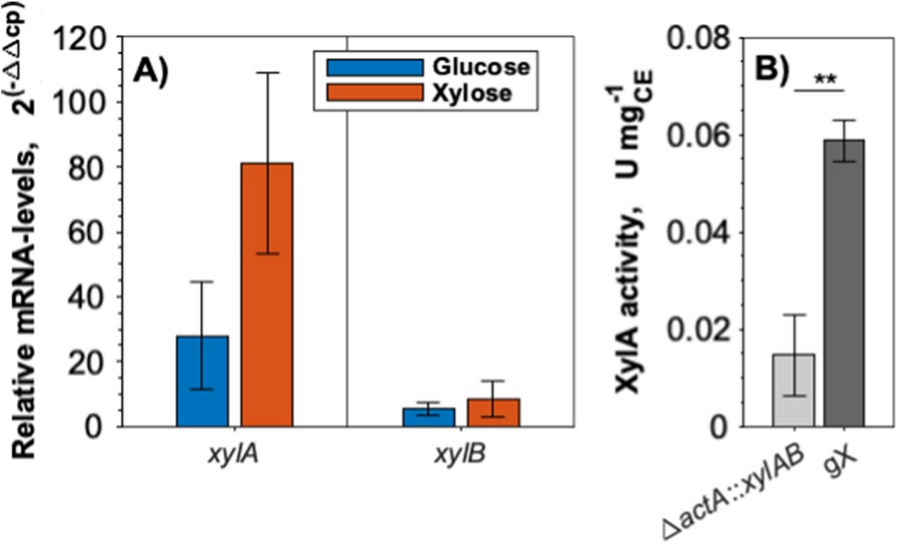
mRNA levels of *xylA* and *xylB* (**A**) and XylA enzyme activity in crude extracts (**B**) of *C. glutamicum* Δ*actA*::*xylAB* and the evolved strain *C. glutamicum* gX. For gene expression analysis by qRT-PCR, cells were grown in CgXII medium with 40 g glucose L^−1^ or 40 g xylose L^−1^ (**A**). Xylose isomerase activity was analyzed in crude extracts of *C. glutamicum* Δ*actA*::*xylAB* and evolved *C. glutamicum* gX, grown in LB (**B**). Significance was determined based on a two-sided unpaired Student’s t-test (**p < 0.01). Data represent means of 3 biological replicates with standard deviations

Next, we studied if this duplication altered the expression of the synthetic *xylAB* operon. Therefore, mRNA levels were quantified by qRT-PCR and compared between the strains *C. glutamicum* gX and *C. glutamicum* Δ*actA*::*xylAB.* Indeed, *xylA* mRNA levels in strain *C. glutamicum* gX were about 80-fold and 28-fold higher than in the parental strain *C. glutamicum* Δ*actA*::*xylAB* when grown with xylose and glucose, respectively (Fig. 6A)*.* The *xylB* mRNA levels in strain gX were increased by eightfold and fourfold compared to the parental strain *C. glutamicum* Δ*actA*::*xylAB* after growth with xylose and glucose, respectively (Fig. 6A)*.* To test if the observed gene expression increase would manifest in increased activity of the *xylA* encoded xylose isomerase, crude extracts of both strains cultivated with either glucose or xylose were prepared and assayed for xylose isomerase activity. Strain *C. glutamicum* gX showed an approximately fourfold higher xylose isomerase activity than the parental strain (Fig. 6B). Thus, the genomic rearrangement by ALE allowed strain *C. glutamicum* gX to grow faster with xylose due to increased expression of *xylA* and *xylB*. Accordingly, an increase of the xylose isomerase activity could be demonstrated by enzyme assays.

### Utilizing *C. glutamicum* gX for the production from xylose

Beyond growth with xylose, it was aimed to demonstrate the applicability of xylose utilization capabilities of *C. glutamicum* gX for production. l-Glutamate was chosen as a representative product because its formation can easily be triggered by adding an elicitor, such as ciprofloxacin [45]. For this purpose, *C. glutamicum* gX was exposed to 8 µg ciprofloxacin mL^−1^ at mid-exponential growth, using 20 g xylose L^−1^ or a sugar mix containing 15 g glucose L^−1^ and 5 g xylose L^−1^. The accumulation of 3.98 ± 0.39 and 3.65 ± 0.20 g glutamate L^−1^ after 48 h proved the suitability of *C. glutamicum* gX for amino acid production from xylose or sugar mixtures (Table 1).

**Table 1:** Growth and production of *C. glutamicum* gX, Dpa1_gX, MePhe5*_gX, and GRLys1Δ*sugR*Δ*ldhA*_gX with xylose as sole or combined carbon source

Strain	C-source	Biomass, g L^−1^	µ_max_, h^−1^	Product	Product titer, g L^−1^	Product yield, g g^−1^
gX	xylose	n.o.*	n.o.*	l-glutamate	4.0 ± 0.4	0.20 ± 0.02
	mix	n.o.*	n.o.*	l-glutamate	3.7 ± 0.2	0.18 ± 0.01
Dpa1_gX	xylose	0.8 ± 0.1	0.07 ± 0.00	DPA	0.5 ± 0.0	0.03 ± 0.00
	mix	1.7 ± 0.4	0.05 ± 0.00	DPA	0.7 ± 0.0	0.03 ± 0.00
MePhe5*_gX	xylose	2.0 ± 0.1	0.07 ± 0.00	NMePhe	1.1 ± 0.1	0.06 ± 0.01
	mix	2.9 ± 0.5	0.04 ± 0.00	NMePhe	1.2 ± 0.0	0.06 ± 0.00
GRLys1 Δ*sugR*Δ*ldhA*_gX	xylose	1.1 ± 0.1	0.05 ± 0.00	l-lysine	6.1 ± 0.9	0.30 ± 0.04
	mix	2.5 ± 0.2	0.12 ± 0.01	l-lysine	8.9 ± 0.6	0.45 ± 0.03

Cultivations were performed in 500-mL shaking flasks or in Duetz plates (MePhe5*_gX) with CgXII medium supplemented with 20 g xylose L^−1^ or 15 g glucose L^−1^ and 5 g xylose L^−1^ as carbon sources for 48 h (l-glutamate) or 72 h (DPA, NMePhe, l-lysine). Data represent means of 3 biological replicates with standard deviations

*n.o. = no growth observed after ciprofloxacin addition

In order to test if the evolved Δ*actA*::*xylAB* locus as present in strains *C. glutamicum* gX is sufficient to enable fast growth and production of other compounds with xylose as the sole carbon source, the locus was amplified and integrated by homologous recombination into the genomes of three different *C. glutamicum* producer strains. The following strains were chosen: Dpa1 for the production of the aromatic dicarboxylate dipicolinic acid (DPA) [77], MePhe5* for the production of the alkylated amino acid *N*-methylphenylalanine (NMePhe) [41], and the genome-reduced l-lysine producer GRLys1Δ*sugR*Δ*ldhA* [61]. The DNA sequence of the Δ*actA*::*xylAB* module (spanning the inserted *xylAB* operon and approximately 550 bp of up- and downstream flanking regions) was amplified from the genomic DNA of gX and used to replace the *actA* locus in the different producer strains. The resulting strains were named Dpa1_gX, MePhe5*_gX, and GRLys1Δ*sugR*Δ*ldhA*_gX, respectively. Indeed, these strains grew well with 20 g xylose L^−1^ as the sole carbon source and produced 0.54 ± 0.03 g L^−1^ DPA, 1.14 ± 0.11 g L^−1^ NMePhe, and 6.1 ± 0.9 g L^−1^ l-lysine, respectively (Table 1). In addition, growth and production using a carbon source mixture composed of 15 g glucose L^−1^ and 5 g xylose L^−1^ was tested. Biomass formation of all three strains increased (by 47 to 109%) compared to cultivations on pure xylose, while surprisingly, xylose alone supported faster growth of Dpa1_gX and MePhe5*_gX than the carbon source mixture. The DPA and l-lysine titers surpassed those from pure xylose by 25% and 74%, respectively, while NMePhe production was comparable (Table 1).

Taken together, the transfer of the evolved Δ*actA*::*xylAB* locus from strain gX to other *C. glutamicum* strains was sufficient to endow them with efficient xylose utilization for growth and production. It manifested the transfer of the gX module as a generally applicable strategy to obtain fast growth and production from xylose.

#### FAL production from wheat straw hydrolysate

For FAL production from wheat straw hydrolysate, the gX module was transferred to *C. glutamicum* Δ*fasR* cg2692_TTG_, which subsequently was transformed with pEKEx2-*maqu2220* to yield strain *C. glutamicum* Δ*fasR* cg2692_TTG_ gX (pEKEx2-*maqu2220*). In CgXII medium with hydrolysate corresponding to 20 g glucose L^−1^ and 195 µM PCA, the reference strain *C. glutamicum* Δ*fasR* cg2692_TTG_ (pEKEx2-*maqu2220*) reached a titer of 681 ± 27 mg_FAL_ L^−1^ (Additional file 1: Figure S5) with a product yield and a volumetric productivity of 0.071 ± 0.004 Cmol Cmol^−1^ and 57 ± 2 mg_FAL_ L^−1^ h^−1^, respectively (Table 2). However, neither product yield nor volumetric productivity were improved by enabling xylose utilization in strain *C. glutamicum* Δ*fasR* cg2692_TTG_ gX (pEKEx2-*maqu2220*). While the titer of 705 ± 77 mg_FAL_ L^−1^ remained comparable to the control strain, the product yield decreased by 18% to 0.058 ± 0.009 Cmol Cmol^−1^. The volumetric productivity decreased comparably (Table 2).

**Table 2:** KPI of *C. glutamicum* Δ*fasR* cg2692_TTG_ (pEKEx2-*maqu2220*) (control), *C. glutamicum* Δ*fasR* cg2692_TTG_ gX (pEKEx2-*maqu2220*) (gXFAL), *C. glutamicum* Δ*fasR* cg2692_TTG_ CgLP12::(P_*tac*_-*pntAB*-T_*rrnB*_) (pEKEx2-*maqu2220*) (*pntAB*) and *C. glutamicum* Δ*fasR* cg2692_TTG_ CgLP12::(P_*tac*_-*pntAB*-T_*rrnB*_) gX (pEKEx2-*maqu2220*) (*pntAB* gX) on hydrolysate

KPI	Strains			
	Control	gXFAL	*pntAB*	*pntAB* gX
C_FAL, Total_|mg L^−1^	681 ± 27	705 ± 77	597 ± 103	748 ± 12
Y_P/S_|Cmol Cmol^−1^	0.071 ± 0.004	0.058 ± 0.009	0.065 ± 0.01	0.075 ± 0.003
Q_P_|mg L^−1^ h^−1^	57 ± 2	47 ± 5	50 ± 9	62 ± 1

Cultivations were conducted in CgXII medium supplemented with 195 µM PCA. The carbon source was provided by hydrolysate, normalized to a concentration of 20 g glucose L^−1^**.** Data represent means of 3 biological replicates ± standard deviations

**Fig. 7:**
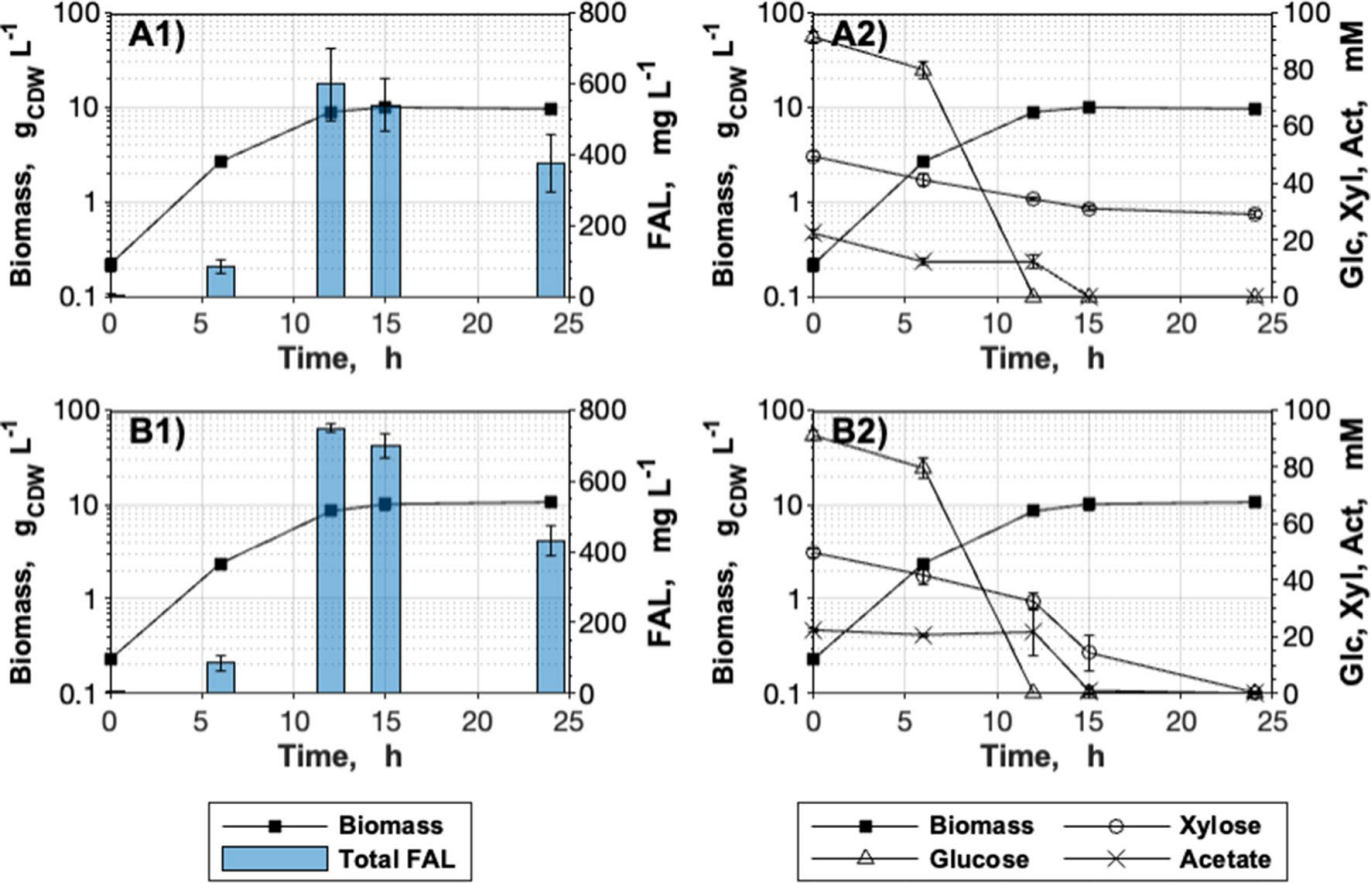
Influence of transhydrogenase expression on FAL production on 2% hydrolysate. Cultivations were conducted in CgXII medium supplemented with 195 µM PCA. The carbon source was provided by hydrolysate, normalized to a concentration of 20 g glucose L^−1^. **A** *C. glutamicum* Δ*fasR* cg2692_TTG_ CgLP12::(P_*tac*_-*pntAB*-T_*rrnB*_)** (**pEKEx2-*maqu2220*); **B** *C. glutamicum* Δ*fasR* cg2692_TTG_ CgLP12::(P_*tac*_-*pntAB*-T_*rrnB*_) gX (pEKEx2-*maqu2220*). Data represent means of 3 biological replicates with standard deviations

Evidently, the majority of carbon supplied by xylose did not end up in the product of interest. Buschke et al. [12] reported insufficient NADPH supply through the oxidative pentose phosphate pathway (PPP) of a modified *C. glutamicum* strain cultivated on xylose. In order to ensure an adequate supply of the cofactor for the NADPH-expensive FAL biosynthesis, the *E. coli* membrane-bound transhydrogenase-encoding genes *pntAB* were genomically integrated into the landing pad CgLP12 [40], resulting in the strain *C. glutamicum* Δ*fasR* cg2692_TTG_ CgLP12::(P_*tac*_-*pntAB*-T_*rrnB*_) gX (pEKEx2-*maqu2220*). The genomic integration of the transhydrogenase increased the volumetric productivity by 32% to 62 ± 1 mg_FAL_ L^−1^ h^−1^ with the gX strain background and increased the product yield by 29% to 0.075 ± 0.003 Cmol Cmol^−1^ (Fig. 7, Table 2). 20% of the 748 ± 12 mg_FAL_ L^−1^ produced after 12 h were measured extracellularly. No positive effects on FAL production upon expressing the transhydrogenase were observed in the control strain *C. glutamicum* Δ*fasR* cg2692_TTG_ CgLP12::(P_*tac*_-*pntAB*-T_*rrnB*_) (pEKEx2-*maqu2220*) lacking the xylose utilization module (Table 2).

Next, we aimed to improve acetyl-CoA availability for FAL production. Therefore, we exchanged the native promotor of *gltA*-encoding citrate synthase in *C. glutamicum* Δ*fasR* cg2692_TTG_ CgLP12::(P_*tac*_-*pntAB*-T_*rrnB*_) gX (pEKEx2-*maqu2220*) for the P_*dapA*_ variants A25, L1 and C7 which reportedly result in a remaining citrate synthase activity of 26, 16 and 10%, respectively [89]. However, FAL production decreased in a promotor strength-dependent manner to 448 ± 11, 277 ± 31, and 254 ± 33 mg_FAL_ L^−1^ for A25, L1, and C7, respectively.

#### Fed-batch cultivation with hydrolysate

**Fig. 8:**
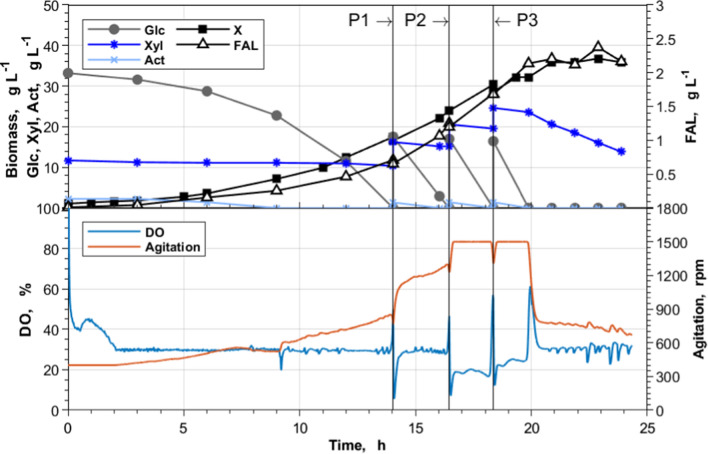
Pulsed fed-batch cultivation of *C. glutamicum* Δ*fasR* cg2692_TTG_ CgLP12::(P_*tac*_-*pntAB*-T_*rrnB*_) gX (pEKEx2-*maqu2220*) with wheat straw hydrolysate. Fed-batch cultivations were conducted in a 1-L BioFlo120^®^ bioreactor system with an initial batch volume of 0.5 L in CgXII_mod_ medium supplemented with 195 µM PCA. The carbon source during the batch phase was provided by hydrolysate, normalized to a concentration of 40 g glucose L^−1^. 30 mL pulses (P) of a 350 g glucose L^−1^ hydrolysate stock solution were added three times upon a sharp increase of the DO signal. Shown process data are representative of three bioreactor cultivations using the same process conditions. *Act* acetate, *DO* dissolved oxygen, *Glc* glucose, *X* biomass, *Xyl* xylose

A scaled-up bioprocess with hydrolysate was conducted using *C. glutamicum* Δ*fasR* cg2692_TTG_ CgLP12::(P_*tac*_-*pntAB*-T_*rrnB*_) gX (pEKEx2-*maqu2220*) as the latest iteration of the FAL-producing strains. Due to the observed sequential xylose utilization, a pulsed feed profile was chosen. The process was conducted three times, and the corresponding KPI characterizing the batch phase, fed-batch phase, and the entire process (total) is listed in Table 3, while process data of one process are shown exemplarily in Fig. 8. During the batch phase, the product yield was on average 42% lower than the yield obtained during the fed-batch phase (0.065 ± 0.008 Cmol Cmol^−1^) (Table 3). Glucose and acetate were always fully consumed during the pulses, while xylose accumulated and was just consumed when glucose was depleted. No noticeable biomass or product formation was observed once xylose consumption started following a linear kinetic. While we encountered extensive foam formation in a glucose-based fed-batch process, the hydrolysate-based process ran very stably. Solely when the glucose provided by the third pulse (Fig. 8, P3) was consumed, foaming occurred. The conducted processes resulted in final FAL titers of 2.45 ± 0.09 g_FAL_ L^−1^ and a volumetric productivity of 0.109 ± 0.005 g_FAL_ L^−1^ h^−1^ (Table 3), which represent a more than threefold and 1.8-fold increase compared to the shaking flask experiments, respectively (Table 2). Notably, compared to shaking flask conditions, over 60% of the overall FAL were secreted into the culture supernatant (data not shown).

**Table 3:** KPI of a pulsed fed-batch process with *C. glutamicum* Δ*fasR* cg2692_TTG_ CgLP12::(P_*tac*_-*pntAB*-T_*rrnB*_) gX (pEKEx2-*maqu2220*) with wheat straw hydrolysate

Phase	KPI	
Batch phase	Q_P_|g L^−1^ h^−1^	0.049 ± 0.001
	Y_P/S_|Cmol Cmol^−1^	0.038 ± 0.002
Fed-batch phase	Q_P_|g L^−1^ h^−1^	0.227 ± 0.028
	Y_P/S_|Cmol Cmol^−1^	0.065 ± 0.008
Total	Q_P_|g L^−1^ h^−1^	0.109 ± 0.005
	Y_P/S_|Cmol Cmol^−1^	0.054 ± 0.005
	CDW_max_|g_X_ L^−1^	37.4 ± 1.1
	c_FAL,max_|g L^−1^	2.45 ± 0.09

The carbon source during the batch phase was provided by hydrolysate, normalized to a concentration of 40 g glucose L^−1^. 30-mL pulses of a 350 g glucose L^−1^ hydrolysate stock solution were added three times upon a sharp increase of the DO signal. Data represent means of 3 biological replicates ± standard deviations

## Discussion

Previously, Takeno et al. [74] demonstrated that deregulation of the FA biosynthetic pathway by inactivating the transcriptional regulator FasR [52] is an effective approach to increase FA synthesis. In contrast to other published studies [33, 74], FA overproduction and efflux relied on nitrogen-limiting cultivation conditions. Accordingly, N-limitation is a common practice for lipid production with several oleaginous organisms [2, 3]. While nitrogen limitation was essential for FA production, FAL synthesis followed a growth-coupled production kinetic improving yield, titer, and volumetric productivity. Similarly, Fillet et al. [25] reported that a high C:N ratio was beneficial for FA production with *Rhodosporidium toruloides*, while a low C:N ratio influenced FAL production positively. However, the underlying molecular mechanisms in *C. glutamicum* still need to be elucidated. As chassis with increased flux through the FA biosynthesis pathway, *C. glutamicum* Δ*fasR* was a suitable basis strain to be engineered for subsequent FAL production. Screening of the two FARs Maqu_2220 and Maqu_2570 of *M. hydrocarbonoclasticus* VT8 without codon optimization revealed that both enzymes were active in *C. glutamicum*. Maqu_2220 was more suited for FAL production by *C. glutamicum* as 35% higher titers were reached with this reductase within 24 h. The observed efficient conversion of long-chain acyl-CoAs into their corresponding alcohols fits with enzymatic studies reporting a high affinity to long-chain acyl-CoA thioesters [32, 85]. Additionally, the obtained FAL distribution agrees with the previously reported FA distribution of *C. glutamicum* [15] and indicates that neither of the reductases had a bias toward one of the three available acyl-CoA substrates.

As reported previously, *C. glutamicum* natively possesses a high thioesterase activity [33, 74]. While reducing this competing side reaction led to an increased FAL production in plasmid-free strains by up to 750%, the introduced start codon exchange had an adverse effect on plasmid-harboring strains. It can be speculated whether the extensive pressure on the acyl-CoA node by a reduced flux towards mycolic acids and additionally high FAR expression and, thus, depletion of acyl-CoAs led to some metabolic burden in those strains. Nevertheless, extracellular FAL titers increased by 120%, and the relative amount of extracellular FAL increased from 12 to 30% in plasmid-harboring TTG start codon mutants. Portevin et al. [55] and Takeno et al. [72] reported an increased permeability of *C glutamicum* upon deleting or downregulating genes directly involved in the mycolic acid biosynthesis downstream of the acyl-CoA node. Attenuating the thioesterase expression thus had a similar effect.

To enable plasmid-free utilization of xylose, we introduced the *xylAB* genes into the locus of the natively highly expressed *actA* gene [62], which was dispensable without notable effects on growth with glucose [78]. The quantitative mRNA analysis displayed higher transcription of *xylAB* in *C. glutamicum* gX, although the promoter sequence and transcriptional start site of replaced *actA* gene [62] were not directly affected by the genomic rearrangement. Therefore, the increased transcription level is obviously the result of an unknown regulatory effect. However, the duplicated region in gX includes the RBS of the originally encoded gene *actA* and resulted in the occurrence of two RBS at a distance of 53 bp, suggesting boosted translation [88].

Xylose utilization by integration of *xylAB* genes into the genome of *C. glutamicum* has been accomplished before. The insertion of one *xylAB* copy under the control of the constitutive *trc* promoter into *C. glutamicum* R, enabled growth with xylose as the sole carbon and energy source [61] but was limited due to low expression levels of both genes, similar to *C. glutamicum* Δ*actA*::*xylAB*. Instead of ALE, Sasaki et al. [61] gradually improved growth by the insertion of up to four additional copies of the *xylAB* genes and elevated the growth rate by 50% from 0.13 to 0.20 h^−1^ [61]. In this study, ALE improved the growth rate by 200% from 0.03 to 0.11 h^−1^. However, further optimization of *xylAB* expression might be beneficial for even faster growth of *C. glutamicum* gX. This might be achieved by the application of fully automated and miniaturized ALE, which was developed and applied to improve the growth rate on xylose 2.6-fold of a *C. glutamicum* strain equipped with the Weimberg pathway for xylose utilization [56]. The genetic changes led to the functional inactivation of the GntR-type transcriptional repressor IolR [56], affecting improved xylose uptake by derepression of the glucose and *myo*-inositol permease IolT1 [9]. Recently, ALE yielded an excellent xylose-utilizing *C. glutamicum* strain, featuring rapid growth (µ_max_ = 0.34 ± 0.00 h^−1^) with xylose [71]. One identified beneficial mutation occurred in an endogenous LacI-type transcriptional regulator involved in inositol metabolism (*ipsA*, cg2910) [5]. Taken together, these findings indicate a close connection between xylose and inositol metabolism and suggest the potential to accelerate xylose utilization of *C. glutamicum* gX by, e.g., derepression of *iolT*1. Moreover, similar to the genomic rearrangement in *C. glutamicum* gX, Sun et al. [71] identified a beneficial point mutation in the P_sod_ promoter of the integrated genes *xylAB*. Additionally, they found and verified a beneficial 21 bp deletion in the 5`UTR promoter region of the genomically integrated xylose importer gene *araE* [71]. Xylose uptake is known as a limiting factor for the efficient utilization of this sugar, as exemplified by various reports on engineering of heterologous importer genes [14, 46, 61, 71, 87]. Therefore, improving xylose uptake in *C. glutamicum* gX seems to be a straightforward approach to advance the growth properties further.

*C. glutamicum* gX turned out to be an excellent glutamate producer and surpassed the reported product yield of 0.13 Cmol Cmol^−1^ (0.13 g g^−1^) from glucose [45] by 40% when xylose served as the sole carbon source. The transfer of the evolved Δ*actA*::*xylAB* module into lysine, DPA, and NMePhe production strains enabled plasmid-free biosynthesis from xylose as sole or with glucose as a combined carbon source. In particular, the product yields for NMePhe and l-lysine of the corresponding strains carrying the gX module with xylose as substrate met or even surpassed previously published yields of plasmid-harboring *C. glutamicum* mutants [41, 53]. Moreover, the successful production of the four compounds mentioned has shown that the implementation of the gX module is generally a promising and transferable approach to make production from xylose-containing second-generation feedstocks, such as lignocellulosic hydrolysates, more sustainable. This was subsequently also demonstrated for FAL, which we produced from refined glucose and wheat straw hydrolysate.

Despite the successfully introduced xylose-utilization module, the majority of carbon provided by xylose and acetate seemed not to end up in FAL. When grown on xylose [12] or acetate [84], *C. glutamicum* exhibits a strongly increased TCA activity, resulting in an increased carbon flux from acetyl-CoA into the TCA. There, a substantial amount of the carbon is oxidized to CO_2_ [12, 84]. For both substrates alike, just a small fraction of carbon is channeled through gluconeogenesis and can thus enter the PPP via the NADPH-regenerating reactions catalyzed by the glucose-6-phosphate and 6-phosphogluconate dehydrogenase [12, 84]. As a consequence, NADPH might become limiting in the NADPH-expensive FAL production. When expressing the NADPH-regenerating *E. coli* transhydrogenase PntAB titers, yields, and volumetric productivities were improved in xylose-utilizing strains. Nevertheless, HPLC data still suggested that product formation was primarily based on carbon provided by glucose, indicating that the precursor supply for FAL synthesis might be limiting when growing on xylose. Reducing the citrate synthase expression as an attempt to increase the acetyl-CoA pool impacted FAL production negatively. Those findings contrast a study by Milke et al. [49], who reportedly improved the malonyl-CoA supply for plant polyphenol production by reducing the citrate synthase expression in a *C. glutamicum* mutant. The same authors simultaneously overexpressed the native acetyl-CoA carboxylase (ACC) [49], which may also be a suitable approach for the here-described FAL producers. Despite deregulated transcription of the ACC’s subunit-encoding genes *accD1* and *accBC* by deletion of *fasR*, the increased flux towards malonyl-CoA might still not be sufficient to fully de-bottleneck the respective pathway.

Even though numerous studies about microbial FAL production have been published using primarily different yeasts [16, 17, 59] or *E. coli* [13, 24, 44], *C. glutamicum* has not been engineered yet for the production thereof. Reported titers and volumetric productivities obtained by oleaginous yeasts range from 98 mg_FAL_ L^−1^ and 1 mg_FAL_ L^−1^ h^−1^ [59] to 5.8 g_FAL_ L^−1^ and 25 mg_FAL_ L^−1^ h^−1^ [16] using *S. cerevisiae* and *Y. lipolytica*, respectively. For FAL production with *E. coli* titers and volumetric productivities of up to 12.5 g_FAL_ L^−1^ and 174 mg_FAL_ L^−1^ h^−1^ are reported [24]. However, respective cultivations with *E. coli* are often conducted in minimal salts media which contain significant concentrations of complex substrates such as yeast extract or tryptone besides the main carbon source [13, 24, 44]. Thus, data about the actual yields, including metabolization of the complex substrates, were rarely provided or solely calculated based on the main substrate’s consumption. Additionally, studies using real second-generation feedstocks like lignocellulosic hydrolysates for FAL production are rare [17], and no such attempts, to our knowledge, have been published with *E. coli* yet. Here, we report a yield, volumetric productivity, and titer of 0.054 ± 0.005 Cmol Cmol^−1^, 0.109 ± 0.005 g_FAL_ L^−1^ h^−1^ and 2.45 ± 0.09 g_FAL_ L^−1^, respectively, which were obtained in a pulsed-fed batch process in a 1-L scale using wheat straw hydrolysate as carbon source. General strain performance can be further improved through targeted and non-targeted approaches focusing on the aforementioned xylose import to improve substrate utilization and the ACC as the bottleneck of the FA biosynthetic pathway. In that context, the constructed final strain *C. glutamicum* Δ*fasR* cg2692_TTG_ CgLP12::(P_*tac*_*-pntAB*-T_*rrnB*_) gX (pEKEx2-*maqu2220*) provides a competitive base for future strain engineering strategies. Therefore, this study does not just represent the first report of FAL production with *C. glutamicum*, but also provides valuable insights into the production thereof from lignocellulosic hydrolysate and the substrate’s metabolization. Furthermore, the organism’s apparent resilience towards high hydrolysate concentrations provides a solid base for sustainable FAL production.

## Conclusion

This study established for the first time de novo production of FAL by *C. glutamicum*, both from a first and a second-generation feedstock. To achieve this, we systematically engineered the FA metabolism and its regulation and optimized the culture conditions. Additional implementation of a transferrable plasmid-free xylose utilization module and further strain optimization enabled sustainable and efficient FAL production from wheat straw hydrolysate. Eventually, a fed-batch process on this second-generation feedstock was established. Therefore, the applied engineering approach and the established bioprocess provide useful principles to optimize FAL production further and utilize *C. glutamicum* as a robust platform for producing other products from wheat straw hydrolysate.

## Materials and methods

### Bacterial strains and plasmids

All strains and plasmids used in this study are listed in Additional file 1: Table S1.

### Media and cultivation

Chemicals used to prepare minimal media were purchased from Carl Roth GmbH & Co. KG (Karlsruhe, Germany). Difco™ tryptone was acquired from Life Technologies Corporation (Detroit, United States), and BBL™ yeast extract was provided by Becton Dickinson GmbH (Heidelberg, Germany).

*E. coli* DH5α was cultivated in 2 × TY [60] medium at 37 °C. If not mentioned otherwise, pre-cultures of *C. glutamicum* strains were cultivated on 2 × TY agar plates containing 18 g agar L^−1^ or in 2 × TY liquid medium, while lysogeny broth (LB; [60] was used for pre-cultures for the xylose isomerase activity assay. Growth experiments were conducted in either a modified version of CgXII minimal medium [10] or a nitrogen-limiting version thereof (NL-CgXII medium). CgXII medium contained per liter 5 g (NH_4_)_2_SO_4_, 5 g urea, 1 g KH_2_PO_4_, 1 g K_2_HPO_4_, 0.25 g MgSO_4_ × 7H_2_O, 0.01 g CaCl_2_, 0.2 mg D-biotin, 0.313 mg CuSO_4_ × 5 H_2_O, 16.4 mg FeSO_4_ × 7 H_2_O, 10 mg MnSO_4_ x H_2_O, 0.02 mg NiCl_2_ × 6 H_2_O, 1 mg ZnSO_4_ × 7 H_2_O. The composition of NL-CgXII medium was similar to CgXII medium, except for the nitrogen source, which was solely provided by 1.45 g urea per liter. The pH was adjusted in both media to 7.4 with 5 N KOH prior to autoclaving. Glucose, xylose, or a corresponding amount of wheat straw hydrolysate (Clariant, Muttenz, Switzerland) were used as carbon sources. Media were supplemented per liter with 50 mg kanamycin, 100 mg spectinomycin, 5 mg tetracycline, and 195 µM PCA when necessary. Gene expression of plasmid-harboring strains was induced by adding 1 mM isopropyl ß-d-1-thiogalactopyranoside (IPTG). *C. glutamicum* was cultivated aerobically in 500 or 100-mL baffled shaking flasks in an orbital shaker (Ø 25 mm, Multitron^®^2, INFORS GmbH, Einsbach, Germany) at 120 rpm and 30 °C, following published seed train procedures [70].

The CgXII medium, used for the production of l-glutamate, l-lysine, dipicolinic acid (DPA) and n-methylphenylalanine (NMePhe), contained 20 g (NH_4_)_2_SO_4_ L^−1^ [22]. For N-NMePhe production, MePhe5*_gX was grown in 24-well Duetz microcultivation plates (Kuhner Shaker GmbH, Herzogenrath, Germany) containing 3 mL CgXII medium per well with 10% nitrogen content (i.e., 0.5 g urea and 2 g ammonium sulfate per liter), supplemented with 0.35 M monomethylamine (MMA) as n-alkyl donor, 0.2 mM l-tryptophan and 0.8 mM l-phenylalanine, l-isoleucine, and L-leucine, respectively.

Glutamate production was elicited by exposure to 8 µg ciprofloxacin mL^−1^ when mid-exponential growth was reached at an OD_600_ of 9 [45].

Strains to be used for cultivations on hydrolysate were pre-cultivated overnight in 50 mL CgXII medium with hydrolysate providing the carbon source at a final concentration of 10 g glucose L^−1^. PCA and kanamycin were added accordingly.

#### Processing of wheat straw hydrolysate

Besides non-quantifiable components, the wheat straw hydrolysate contained primarily glucose, xylose, and acetic acid at concentrations of 380, 150, and 24 g L^−1^, respectively. To process the acidic hydrolysate, an adequate amount thereof was diluted with deionized water, and the pH was set to 7 using 5 N KOH. The final volume was adjusted to obtain a stock solution containing either 125 g glucose L^−1^ or 350 g glucose L^.1^. Precipitated solids of the 125 g glucose L^−1^ stock were removed after autoclaving by centrifugation at 4000 ×*g* for 20 min. The particulate-free supernatant was decanted into a new vessel and used for all shaking flask experiments. The 350 g glucose L^−1^ stock solution was solely centrifuged 4000 ×*g* for 20 min to precipitate solids after adjusting the pH and was used without autoclaving in all bioreactor cultivations.

### Recombinant DNA work

All enzymes used in this study, except for ALLin^TM^ HiFi DNA Polymerase (highQu GmbH, Kraichtal, Germany) and T4 DNA ligase (Promega, Walldorf, Germany), were purchased from New England BioLabs GmbH (Frankfurt am Main, Germany). All enzymes were used in accordance with the manufacturer’s instructions. Genomic DNA, PCR products, and linearized and circular plasmids were purified using the following commercial kits from MACHEREY-NAGEL GmbH & Co. KG (Düren, Germany) according to the manufacturer’s instructions: NucleoSpin^®^ Microbial DNA, NucleoSpin^®^ Gel and PCR Clean-up, NucleoSpin^®^ Plasmid. Oligonucleotides were purchased from Sigma-Aldrich Chemie GmbH (Taufkirchen, Germany) and are listed in Additional file 1: Table S2. All relevant sequences of newly constructed plasmids and genomic integrations were verified via Sanger-Sequencing conducted by Microsynth Seqlab GmbH (Göttingen, Germany). Deletions were confirmed using colony-PCR. Genomic DNA of *Marinobacter hydrocarbonoclasticus* VT8 (DSM No.: 11845), purchased from the German Collection of Microorganisms and Cell Cultures GmbH (Braunschweig, Germany), was used as a template to amplify *maqu_2220* and *maqu_2507*. The corresponding oligonucleotides required for the amplification of the FAR genes were designed to contain an artificial RBS (GAAAGGAGA) with a corresponding spacer (GGATTG) [81] upstream of their start codons.

*E. coli* DH5α was used as a cloning strain to amplify and maintain plasmids. DNA fragments and genes were either amplified with Phusion^®^ High-Fidelity DNA Polymerase or with ALLin^™^ HiFi DNA Polymerase. Chromosomal modifications of *C. glutamicum* were conducted by double homologous cross-over events using the pK19*mobsacB* vector system [72]. Purified DNA fragments and linearized plasmids were joined via Gibson Assembly [28]. pK19*mobsacB*_Δ*actA*::*xylAB* (P5) was assembled via Golden Gate cloning with *Bsa*I as type IIS restriction enzyme and T4 DNA ligase [23]. Therefore, pK19*mobsacB* was modified to be suitable for the modular assembly of multiple DNA fragments as described elsewhere [81], yielding pK19*mobsacB*_GG (P2). pEKEx2 was standardly linearized with *Eco*RI, while pK19*mobsacB* was primarily cut with *Sma*I. Solely, the construction of plasmids P3 and of P9 required the use of *Nhe*I and *Bam*HI, respectively. The batches containing the assembled products were used to transform chemically competent *E. coli* DH5α [60] via heat-shock at 42 °C. Correctly assembled plasmids were subsequently used to transform electrocompetent *C. glutamicum* via electroporation, followed by a heat shock at 46 °C [75, 76].

### Bioreactor cultivation

Fed-batch cultivations were conducted in a 1 L BioFlo120^®^ bioreactor system (Eppendorf SE, Hamburg, Germany) with an initial volume of 0.5 L. The cultivations were performed at 30 °C using an adjusted version of the CgXII medium to obtain high biomass concentrations (CgXII_mod_). The medium contained per liter 40 g ammonium sulfate, 2 g KH_2_PO_4_, 2 g K_2_HPO_4_, 1 g MgSO_4_ × 7H_2_O, 0.01 g CaCl_2_, 0.2 mg D-biotin, 0.626 mg CuSO_4_ × 5 H_2_O, 32.8 mg FeSO_4_ × 7 H_2_O, 20 mg MnSO_4_ × H_2_O, 0.04 mg NiCl_2_ × 6 H_2_O, 2 mg ZnSO_4_ × 7 H_2_O. Kanamycin, PCA, and IPTG were supplemented as described above. The batch phase was conducted with a glucose equivalent per liter of 40 g glucose obtained by the addition of the prepared wheat straw hydrolysate. 30 mL of a 350 g glucose L^−1^ hydrolysate stock solution was repeatedly pulsed into the bioreactor upon depletion of the carbon source, as indicated by a spike of the DO. The pH was set to 7.4 using a 5 M KOH solution. Dissolved oxygen (DO) was kept above 30% by adjusting the agitation between 400 and 1500 rpm, with a constant air inflow rate of 0.25 L min^−1^ (0.5 vvm). After adding the third hydrolysate pulse, the aeration rate was increased to 0.4 L min^−1^. Agitation was provided by a 6-bladed Rushton-type impeller. A constant feed of the antifoaming agent CONTRASPUM^®^ A 4050 (Zschimmer & Schwarz GmbH & Co KG, Lahnstein, Germany) with a feed rate of 100 µL h^−1^ was started between 9 and 11 h and was kept until the end of the respective process. Manual addition of the antifoaming agent was done aseptically when required.

### Analytics

#### HPLC analysis

Glucose, xylose, pyruvate, lactate, and acetate were quantified via high-performance liquid chromatography (HPLC) using an Agilent 1260 Infinity II system (Agilent Technologies, Waldbronn, Germany) equipped with a Hi-Plex H column (7.7 × 300 mm, 8 µm) and a Hi-Plex H guard cartridge (3 × 5 mm, 8 µm). Samples were isocratically eluted at 50 °C for 35 min using 5 mM H_2_SO_4_ as a mobile phase with a flow rate of 0.4 mL min^−1^. Analytes were detected via a refractive index detector (RID) kept at 50 °C [70].

Dicarboxylate dipicolinic acid (DPA) was analyzed by an amino exchange column (Aminex, 8 × 300 mm, 10 µm, 25 Å pore diameter, CS Chromatographie Service GmbH, Langerwehe, Germany), using 5 mM H_2_SO_4_ at a flow rate of 0.8 mL min^−1^ for 30 min under isocratic conditions [65] and detected by a RID.l-Glutamate, l-lysine and n-methylphenylalanine (NMePhe) were separated via reversed-phase HPLC, equipped with a pre- and a main-column (LiChrospher 100 RP18 EC-5 m (40 × 4 mm) and LiChrospher 100 RP18 EC-5 m (125 × 4 mm), CS Chromatographie Service GmbH, Langerwehe, Germany) and detected by a fluorescence detector (FLD G1321A, 1200 series, Agilent Technologies, Deutschland GmbH, Böblingen, Germany). l-glutamate and l-lysine were derivatized with ortho-phthaldialdehyde (OPA) [66] and detected at 230 nm excitation and 450 nm emission wavelengths with l-asparagine as internal standard.

NMePhe was detected upon derivatization with fluorenylmethyl chloroformate (FMOC) (Karl Roth, Karlsruhe, Germany) [64]. The separation was performed at a flow rate of 1.2 mL min^−1^, using sodium acetate (50 mM, pH 4.2) (A) and acetonitrile (B) as eluents at a gradient of: 0 min 38% B, 5 min 38% B, 10 min 48% B, 12 min 52% B, 13 min 57% B, 14 min 63% B, 16 min 68% B, 17 min 76% B and 20 min 38% B and fluorescence was detected at 250 nm excitation and 410 nm emission wavelengths with l-proline as internal standard [41].

#### Quantification of fatty alcohols and fatty acids

Gas chromatography was used to quantify fatty alcohols (FAL) and fatty acids (FA), the latter in the derivatized form of fatty acid methyl esters (FAMEs). All samples were analyzed on an Agilent 8890 GC system (Agilent Technologies, Waldbronn, Germany) equipped with an Agilent DB-FATWAX-UI GC column (30 m × 0.25 mm × 0.25 µm). The injected sample volume was 1 µL with a split ratio of 10:1 for FAL and extracellular FA samples. A split ratio of 50:1 was applied for intracellular FA samples. The initial oven temperature was 90 °C for 0.5 min, followed by a 40 °C min^−1^ ramp to 165 °C, held for 1 min, and a final 3 °C min^−1^ ramp to 230 °C, held for 10 min. The method was operated with a constant flow of 0.89 mL min^−1^ of the carrier gas nitrogen. The flame ionization detector (FID) and the inlet were maintained at 280 °C and 250 °C, respectively.

FALs were extracted and analyzed similarly to previously described methods [13, 16, 82]. Briefly, 0.5 mL of either supernatant or culture broth were mixed with heptadecanol as an internal standard (ISTD) with a final concentration of 50 µg mL^−1^ and were extracted with 0.5 mL ethyl acetate for 30 min under vigorous vortexing. The organic phase was dried over sodium sulfate and subsequently used for GC-FID analysis to determine extracellular and total FAL concentrations. Intracellular FAL concentrations were obtained by subtracting extracellular from total FAL concentrations.

Extracellular FA were extracted from 0.8 mL supernatant containing 62.5 µg heptadecanoic acid mL^−1^ as ISTD by the Bligh-Dyer method [7]. 1 mL of the lipid-containing organic phase was evaporated under vacuum using a Concentrator plus (Eppendorf SE, Hamburg, Germany). The dried extracts were re-suspended in 0.5 mL methanol and transferred into a glass tube with a PTFE-lined screw cap. Acid-catalyzed methylation was achieved by adding 1 mL of 5% (v/v) methanolic H_2_SO_4_ to the samples and incubating them for 2 h in a water bath at 95 °C. After cooling to room temperature, 0.5 mL hexane was added, and the samples were vortexed vigorously. The acidic solution was neutralized by adding 2.5 mL aqueous 6% (w/v) NaHCO_3_, before transferring the hexane layer into a GC vial for analysis.

Intracellular fatty acids were analyzed based on an acid-catalyzed whole-cell transesterification described previously [54]. Cell pellets obtained from 1 mL of cell culture were washed with 3 mL saline and subsequently dried at 70 °C before adding 50 µg heptadecanoic acid as an ISTD. 1 mL of 5% (v/v) methanolic H_2_SO_4_ was added, and the samples were subjected to transesterification at 95 °C for 2 h. FAMEs were extracted with hexane using the same procedure applied for extracellular FA samples and were subjected to analysis.

FAL were quantified using authentic 1-hexadecanol, 1-octadecanol and oleyl alcohol standards, while fatty acids were quantified using palmitic and oleic acid standards. Relative FA content was defined as the ratio of the intracellular FA concentration to cell dry weight (CDW) when applicable.

#### Total organic carbon

The total organic carbon (TOC) content of hydrolysate-containing supernatants was determined as described previously [10] by using a Multi N/C 2100s analyzer (Analytik Jena, Jena, Germany). Briefly, to determine a sample’s total carbon (TC) content, 100 µL culture supernatant were injected and combusted at 800 °C. The total inorganic carbon (TIC) content was obtained by injecting 100 µL of the sample, which was subsequently acidified with 10% (w/v) o-phosphoric acid. CO_2_ released by both procedures was measured by a nondispersive infrared sensor and used to derive the corresponding carbon content. The measured signals were converted into a carbon concentration in g_C_ L^−1^ by applying calibration curves obtained from sodium carbonate and potassium hydrogen phthalate standard mixtures in the range of 0.1–1.5 g_C_ L^−1^ (TIC) and 0.4–3 g_C_ L^−1^ (TC), respectively. The TOC was obtained by subtracting the TIC from TC.

#### Determination of growth parameters

Growth during shaking flask and bioreactor cultivations was monitored by measuring the optical density at 600 nm (OD_600_) with an Ultrospec 10 cell density meter (Harvard Biochrom, Holliston, MA, USA). A previously determined correlation factor of 0.23 [63] was used to convert OD_600_ to a biomass concentration in g cell dry weight (CDW) L^−1^. The growth rate µ, biomass yield Y_X/S_, product yield Y_P/S_ and biomass-specific glucose consumption rate q_S_ were determined as described elsewhere [31]. The Y_P/S_ for cultivations on hydrolysate was calculated differentially based on the total organic carbon consumed between t = 0 h and the sampling point corresponding to the highest measured FAL titer. Extracellular FAL concentrations were taken into account, and the corresponding carbon concentration was subtracted from the TOC data before forming the quotient. Experimental data were obtained from at least three independent biological replicates with individual seed trains. If not stated otherwise, data represent means of ≥ 3 biological replicates with respective standard deviations.

#### Gene expression analysis by qRT-PCR

Gene expression was analyzed by mRNA levels via quantitative reverse transcription PCR (qRT-PCR). Strains were grown in CgXII medium supplemented per liter with 40 g glucose or xylose in triplicates and harvested at OD_600_ = 5 by centrifugation (15 s, 14,000 ×*g*). Pellets were immediately frozen in liquid nitrogen and stored at −80 °C until RNA isolation.

For RNA isolation, cell pellets were homogenized by resuspension in 100 µL TE buffer (10 mM Tris–HCL, 1 mM EDTA, pH 8), containing 5 mg mL^−1^ lysozyme for 30 min at 37 °C. Total RNA was extracted with the NucleoSpin^®^ RNA kit (Macherey‐Nagel, Düren, Germany) and subsequently treated with RNase‐free DNase Set and RNeasy MinElute kits (Qiagen, Hilden, Germany) according to the manufacturers` protocols. RNA concentrations were determined by spectrophotometry (NanoDrop^®^, ND‐1000; ThermoFisher Scientific, Schwerte, Germany), and agarose gel electrophoresis was performed for quality control of RNA purity and integrity.qRT-PCR samples were prepared using the SensiFAST^™^ SYBR^®^ No‐ROX One‐Step Kit (Bioline, London, UK) according to the manufacturer. qRT-PCR was performed in a CFX96 cycler system (Bio‐Rad, Hercules, CA, USA) with a temperature profile of 45 °C for 10 min, 95 °C for 2 min, and 40 cycles of 95 °C for 5 s, 56 °C for 15 s, and 72 °C for 15 s and melt curve analysis between 65 and 95 °C. Expression levels were calculated by the ΔC_q_ method [30].

#### Xylose isomerase activity assay

Strains were cultivated overnight in LB, harvested by centrifugation (20,200 ×*g*, 7 min) at 4 °C, washed, and resuspended in 2 mL TRIS–HCl buffer (100 mM, pH 7.5). Cells were disrupted by sonication (UP 200S, Dr. Hielscher GmbH, Teltow, Germany) at 60% amplitude and a duty cycle of 0.5 s for 9 min. The method of Bradford [8] was used to determine the total protein concentrations of crude extracts of *C. glutamicum* Δ*actA*::*xylAB* and gX, respectively. A bovine serum standard served as a reference. Sample preparation and xylose isomerase activity measurements were performed as described elsewhere [29]. The assay was performed with 30 µL crude extract and 30 U sorbitol dehydrogenase per reaction in triplicates. The specific activity in U min^−1^ was defined as µmol min^−1^ (mg protein)^−1^. Significance of the xylose isomerase assay was determined based on a two-sided unpaired Student's t-test.

#### Adaptive laboratory evolution and whole-genome sequencing

ALE of *C. glutamicum* was performed in 100 mL baffled shaking flasks, using CgXII medium supplemented with 40 g xylose L^−1^ as the sole carbon source. An LB overnight culture of a single colony was used to inoculate the first main culture, followed by serial batch cultivations, all starting at an OD_600_ of 1. When OD_600_ > 2 was reached after 24 h or more, the cells were transferred to fresh CgXII medium until an accelerated µ and an increased total biomass formation were observed in the fifth batch. From this culture, single colonies of the mutant, designated as *C. glutamicum* gX, were isolated, of which three were analyzed by whole-genome sequencing.

Genomic DNA of the strain *C. glutamicum* Δ*actA*::*xylAB* and the three isolated colonies of the evolved mutant strain *C. glutamicum* gX was isolated from LB overnight cultures using the NucleoSpin Microbial DNA kit for DNA, RNA, and protein purification (Macherey–Nagel, Düren, Germany) according to the manufacturer. The purity of genomic DNA was confirmed by spectrophotometry (NanoDrop^®^, ND-1000), and complete RNA digestion was verified by gel electrophoresis.

For whole-genome sequencing, samples were prepared using the Illumina TruSeq DNA PCR-free library prep kit (Illumina, San Diego, USA), according to the manufacturer. A MiSeq sequencer system 2 × 300 nt PE (Illumina, San Diego, USA) was used to perform Illumina genome sequencing, and raw sequencing data are available as Bioproject PRJNA895044. NGS raw reads were trimmed and mapped using Bowtie2 [41] paired-end mode with standard settings and *C. glutamicum* ATCC 13032 (CP025533) modified with the introduced mutations Δ*actA*::*xylAB* as the reference genome.

SNP detection of mapped sequencing data with 90% minimum percentage of variation as minimum was carried out using snippy v.4 [68].

For additional analysis by Nanopore sequencing technology, libraries were prepared using the ONT SQK-LSK109 ligation sequencing kit (Oxford Nanopore Technologies Oxford, UK). The long read sequencing data, generated on the ONT GridION platform with an R9.4.1 flow cell (Oxford Nanopore Technologies Oxford, UK), was basecalled and demultiplexed with guppy v4.0.11. For assembly, canu v.2.1.1 (parameters: genome Size = 3.5 m, raw Error Rate = 0.3, corrected Error Rate = 0.1) [37] was used. The assembled genomes were polished using Racon v1.3.3 (parameters: − c 6, − m 8, − x − 6, − g − 8, − w 500) [77], Medaka v1.2.3 (parameters: − b 100, − m r941_min_high_g303) (Oxford Nanopore technologies, 2020) and Pilon v1.22 [80]. Genome comparison of the resulting assemblies was performed with mauve [19].

## Supplementary Information

**Additional file 1: Table S1.** Strains and plasmids used in this study. **Table S2.** Oligonucleotides used in this study. Complementary overlaps are italicized, and synthetic RBS + spacer sequences, as described by Shi et al. [8], are displayed in bold. **Figure S1.** Influence of *fasR *deletion on fatty acid production under nitrogen-limiting conditions. Cultivations were conducted in the nitrogen-limiting NL-CgXII medium containing per liter 20 g glucose as the carbon source. **A** *C. glutamicum* WT; **B** *C. glutamicum* Δ*fasR*. Data represent means of ≥ 3 biological replicates with standard deviations. **Figure S2.** Fatty alcohol production of plasmid-harboring and plasmid-free *C. glutamicum* Δ*fasR* mutants with the FAR Maqu2220. Cultivations were conducted in NL-CgXII medium containing 20 g glucose L^-1^. Samples for FAL analysis were taken after 48 h. Data represent means of 3 biological replicates with standard deviations. **Figure S3.** ALE of *C. glutamicum* Δ*actA*::*xylAB* in shaking flasks in 6 serial batch cultivations over 382 h in total, using CgXII medium containing 40 g xylose L^-1^ as sole carbon source. Transfers to fresh CgXII medium were performed when OD_600_ surpassed at least 2 after 24 h or more. **Figure S4.** ALE of *C. glutamicum* Δ*actA*::*xylAB* on xylose. **A** Growth curves of *C. glutamicum* Δ*actA*::*xylAB *and the evolved strain gX. Cultivations were conducted in CgXII medium containing 40 g xylose L^-1^ as the sole carbon source. Data represent means of 3 biological replicates with standard deviations.** B** Nucleotide sequence of the 5’ untranslated regions (UTR) of *C. glutamicum* Δ*actA*::*xylAB* and *C. glutamicum* gX. The duplicated region, comprising 21 bp of the 5’UTR and 32 bp of the beginning of xylose isomerase gene *xylA*, is highlighted (blue box) in the parental strain *C. glutamicum* Δ*actA*::*xylAB* and in the evolved strain gX. The putative RBS [62] is indicated in bold letters, and the start codon of *xylA* is highlighted in green. **Figure S5.** FAL production on wheat straw hydrolysate. Cultivations were conducted in CgXII medium supplemented with 195 µM PCA. The carbon source was provided by hydrolysate, normalized to a concentration of 20 g glucose L^-1^. **A** *C. glutamicum* Δ*fasR* cg2692_TTG_ (pEKEx2-*maqu2220*); **B** *C. glutamicum* Δ*fasR* cg2692_TTG_ gX (pEKEx2-*maqu2220*). Data represent means of 3 biological replicates with standard deviations.

## Data Availability

All data generated or analyzed during this study are included in this published article and its supplementary information file.
