# Supplementary material for: Metabolic engineering of Corynebacterium glutamicum for fatty alcohol production from glucose and wheat straw hydrolysate
Source: Biotechnol Biofuels Bioprod. 2023 Jul 18;16:116. doi: 10.1186/s13068-023-02367-3 (PMC10355004; doi:10.1186/s13068-023-02367-3)
Supplement: Supplementary file 1 — Additional file 1: Table S1. Strains and plasmids used in this study. Table S2. Oligonucleotides used in this study. Complementary overlaps are italicized, and synthetic RBS + spacer sequences, as described by Shi et al. [8], are displayed in bold. Figure S1. Influence of fasR deletion on fatty acid production under nitrogen-limiting conditions. Cultivations were conducted in the nitrogen-limiting NL-CgXII medium containing per liter 20 g glucose as the carbon source. A C. glutamicum WT; B C. glutamicum ΔfasR. Data represent means of ≥ 3 biological replicates with standard deviations. Figure S2. Fatty alcohol production of plasmid-harboring and plasmid-free C. glutamicum ΔfasR mutants with the FAR Maqu2220. Cultivations were conducted in NL-CgXII medium containing 20 g glucose L-1. Samples for FAL analysis were taken after 48 h. Data represent means of 3 biological replicates with standard deviations. Figure S3. ALE of C. glutamicum ΔactA::xylAB in shaking flasks in 6 serial batch cultivations over 382 h in total, using CgXII medium containing 40 g xylose L-1 as sole carbon source. Transfers to fresh CgXII medium were performed when OD600 surpassed at least 2 after 24 h or more. Figure S4. ALE of C. glutamicum ΔactA::xylAB on xylose. A Growth curves of C. glutamicum ΔactA::xylAB and the evolved strain gX. Cultivations were conducted in CgXII medium containing 40 g xylose L-1 as the sole carbon source. Data represent means of 3 biological replicates with standard deviations. B Nucleotide sequence of the 5’ untranslated regions (UTR) of C. glutamicum ΔactA::xylAB and C. glutamicum gX. The duplicated region, comprising 21 bp of the 5’UTR and 32 bp of the beginning of xylose isomerase gene xylA, is highlighted (blue box) in the parental strain C. glutamicum ΔactA::xylAB and in the evolved strain gX. The putative RBS [62] is indicated in bold letters, and the start codon of xylA is highlighted in green. Figure S5. FAL production on wheat straw hydrolysate. Cultivations w [file 13068_2023_2367_MOESM1_ESM.docx]

Additional file

Table S1: Strains and plasmids used in this study

| - **No.** | - **Strain or plasmid** | - **Relevant characteristics** | - **Reference** |
| --- | --- | --- | --- |
|  | - **Strains** |  |  |
| - S1 | - *E. coli* DH5α | - F-Φ80*lacZ*ΔM15 Δ(*lacZYA-argF*) U169 *endA1 recA1* *hsdR17* (rK^-^, mK^+^) *supE44* *thi-1 gyrA96 relA1 phoA* | - (Hanahan, 1983) |
| - S2 | - *E. coli* K-12 MG1655 | - Wild type (WT) | - (Blattner et al., 1997) |
| - S3 | - *C. glutamicum* ATCC 13032 | - Wild type (WT) | - (Abe et al., 1967; Kinoshita et al., 1958) |
| - S4 | - *C. glutamicum* Δ*fasR* | - *C. glutamicum* ATCC 13032 derivate with deletion of the transcriptional regulator-encoding gene *fasR* | - This study |
| - S5 | - *C. glutamicum* Δ*fasR* cg2692_GTG_ | - *C. glutamicum ∆fasR* derivate with start codon GTG of the thioesterase-encoding gene cg2692 | - This study |
| - S6 | - *C. glutamicum* Δ*fasR* cg2692_TTG_ | - *C. glutamicum ∆fasR* derivate with start codon TTG of the thioesterase-encoding gene cg2692 | - This study |
| - S7 | - *C. glutamicum* Δ*fasR* CgLP11::(P*_tac_*-*maqu2220*-T*_rrnB_*) | - *C. glutamicum ∆fasR* derivate with *maqu_2220* of *M.* *hydrocarbonoclasticus* VT8, under control of P*_tac_* and terminated by T*_rrnB_*, integrated into landing pad CgLP11 | - This study |
| - S8 | - *C. glutamicum* Δ*fasR* CgLP11::(P*_tac_*-*maqu2220*-T*_rrnB_*) cg2692_GTG_ | - *C. glutamicum* Δ*fasR* CgLP11::(P*_tac_*-*maqu_2220*-T*_rrnB_*) derivate with start codon GTG of the thioesterase-encoding gene cg2692 | - This study |
| - S9 | - *C. glutamicum* Δ*fasR* CgLP11::(P*_tac_*-*maqu2220*-T*_rrnB_*) cg2692_TTG_ | - *C. glutamicum* Δ*fasR* CgLP11::(P*_tac_*-*maqu_2220*-T*_rrnB_*) derivate with start codon TTG of the thioesterase-encoding gene cg2692 | - This study |
| - S10 | - *C. glutamicum* Δ*actA*::*xylAB* | - *C. glutamicum* ATCC 13032 derivate with the synthetic operon, consisting of *xylA* of *Xanthomonas campestris* and *xylB* of *C. glutamicum* WT, integrated into the gene locus *actA* (cg2840) | - This study |
| - S11 | - *C. glutamicum* gX | *C. glutamicum* Δ*actA*::*xylAB* derivate with evolved sequence upstream of *xylA* | - This study |
| - S12 | - *C. glutamicum* Δ*fasR* cg2692_TTG_ gX | - *C. glutamicum* Δf*asR* cg2692_TTG_ derivate with insertion of the synthetic *xylAB* operon, harboring the evolved sequence upstream of *xylA*, integrated into the gene locus *actA* | - This study |
| S13 | *C. glutamicum* Δ*fasR* cg2692_TTG_ CgLP12::(P*_tac_*-*pntAB*-T*_rrnB_*) | *C. glutamicum* Δf*asR* cg2692_TTG_ derivate with *pntAB* of *E. coli*, under control of P*_tac_* and terminated by T*_rrnB_*, integrated into landing pad CgLP12 | This study |
| S14 | *C. glutamicum* Δ*fasR* cg2692_TTG_ CgLP12::(P*_tac_*-*pntAB*-T*_rrnB_*) gX | *C. glutamicum* Δf*asR* cg2692_TTG_ gX derivate with *pntAB* of *E. coli*, under control of P*_tac_* and terminated by T*_rrnB_*, integrated into landing pad CgLP12 | This study |
| S15 | - *C. glutamicum* Δ*fasR* cg2692_TTG_ CgLP12::(P*_tac_*-*pntAB*-T*_rrnB_*) ΔP*gltA*::P*dapA*-A25 gX | *C. glutamicum* Δ*fasR* cg2692_TTG_ CgLP12::(P*_tac_*-*pntAB*-T*_rrnB_*) gX with *gltA* promotor exchanged for the *dapA* promotor variant A25 | This study |
| S16 | *C. glutamicum* Δ*fasR* cg2692_TTG_ CgLP12::(P*_tac_*-*pntAB*-T*_rrnB_*) ΔP*gltA*::P*dapA*-C7 gX | *C. glutamicum* Δ*fasR* cg2692_TTG_ CgLP12::(P*_tac_*-*pntAB*-T*_rrnB_*) gX with *gltA* promotor exchanged for the *dapA* promotor variant C7 | This study |
| S17 | *C. glutamicum* Δ*fasR* cg2692_TTG_ CgLP12::(P*_tac_*-*pntAB*-T*_rrnB_*) ΔP*gltA*::P*dapA*-L1 gX | *C. glutamicum* Δ*fasR* cg2692_TTG_ CgLP12::(P*_tac_*-*pntAB*-T*_rrnB_*) gX with *gltA* promotor exchanged for the *dapA* promotor variant L1 | This study |
| S18 | *C. glutamicum* GRLys1Δ*sugR*Δ*ldhA* | *C. glutamicum* WT Δ*pck*, *pyc*^P458S^, *hom*^V59A^, 2 copies of *lysC*^T311I^, 2 copies of *asd*, 2 copies of *dapA*, 2 copies of *dapB*, 2 copies of *ddh*, 2 copies of *lysA*, 2 copies of *lysE*, in-frame deletion of prophages CGP1, CGP2 and CGP3, Δ*sugR*Δ*ldhA* | (Pérez-García et al., 2016) |
| S19 | *C. glutamicum* GRLys1Δ*sugR*Δ*ldhA_*gX | *C. glutamicum* GRLys1Δ*sugR*Δ*ldhA* derivate with insertion of the synthetic operon *xylAB* with evolved sequence upstream of *xylA* as in gX integrated into the gene locus *actA* | This study |
| S20 | *C. glutamicum* Dpa1 | *C. glutamicum* WT *pyc*^P458S^, *hom*^V59A^, *lysC*^T311I^Δ*pck* carrying pECXT_P_syn_-*dpaAB* | (Schwardmann et al., 2022) |
| S21 | *C. glutamicum* Dpa1_gX | *C. glutamicum* Dpa1 derivate with insertion of the synthetic operon *xylAB* with evolved sequence upstream of *xylA* as in gX integrated into the gene locus *actA* | This study |
| S22 | *C. glutamicum* MePhe5* | *C. glutamicum* WT Δ*qsuBCD*::P*_tuf_*-*qsuC*Δ*ppc*::P*_sod_*-*aroB*ΔP*_tkt_*::P*_tuf_*-*tkt*Δ*iolR*::P*_tuf_*-*aroE*Δt*rpEG*Δ*ilvE*Δ*aroT* carrying pEKEx3_*pheA*^FBR^ and pVWEx1_ pVWEx1_*dpkA*^P262M141L^ | (Kerbs et al., 2021) |
| S23 | *C. glutamicum* MePhe5*_gX | *C. glutamicum* MePhe5* derivate with insertion of the synthetic operon *xylAB* with evolved sequence upstream of *xylA* as in gX integrated into the gene locus *actA* | This study |
|  | - **Plasmids** |  |  |
| - P1 | - pK19*mobsacB* | - Km^r^; Mobilizable cloning vector to construct insertion or deletion mutants of *C. glutamicum* (pK18 *ori*V*_E.c_*_._, *sacB*, *lacZα*) | - (Schäfer et al., 1994) |
| - P2 | - pK19*mobsacB*_GG | - Km^r^; pK19*mobsacB* derivative with fusion sites and BsaI recognition site for Golden Gate assembly | - This study |
| - P3 | - pK19*mobsacBlacI^q^* | - Km^r^; pK19*mobsacB* derivate with *lacI*^q^ downstream of the multiple cloning site (MCS) | - This study |
| - P4 | - pK19*mobsacB*_ Δ*fasR* | - Km^r^; pK19*mobsacB* derivate for the deletion of *fasR* | - This study |
| - P5 | - pK19*mobsacB*_Δ*actA*::*xylAB* | - Km^r^; pK19*mobsacB* derivate for the deletion of *actA* and the integration of genes encoding XylA (xylose isomerase) of *Xanthomonas campestris* and XylB (xylulokinase) of *C. glutamicum* into the *actA* locus | - This study |
| P6 | pK19*mobsacB*_ ΔP*gltA*::P*dapA*-A25 | Km^r^; pK19*mobsacB* derivate for exchanging the native promotor of *gltA* for the *dapA* promotor variant A25 | (van Ooyen et al., 2012) |
| P7 | pK19*mobsacB*_ ΔP*gltA*::P*dapA*-C7 | Km^r^; pK19*mobsacB* derivate for exchanging the native promotor of *gltA* for the *dapA* promotor variant C7 | (van Ooyen et al., 2012) |
| P8 | pK19*mobsacB*_ ΔP*gltA*::P*dapA*-L1 | Km^r^; pK19*mobsacB* derivate for exchanging the native promotor of *gltA* for the *dapA* promotor variant L1 | (van Ooyen et al., 2012) |
| - P9 | - pK19*mobsacB*_gX | Km^r^; pK19*mobsacB* derivate for the deletion of *actA* and the integration of *xylAB* with evolved sequence upstream of *xylA* as in strain gX into the *actA* locus | - This study |
| - P10 | - pK19*mobsacB*_cg2692_GTG_ | - Km^r^; pK19*mobsacB* derivate for exchanging the native start codon of cg2692 to GTG | - This study |
| - P11 | - pK19*mobsacB*_cg2692_TTG_ | - Km^r^; pK19*mobsacB* derivate for exchanging the native start codon of cg2692 to TTG | - This study |
| - P12 | - pK19*mobsacBlacI^q^*_ *maqu2220*_CgLP11_ | - Km^r^; pK19*mobsacBlacI^q^* derivate for the integration of *maqu_2220*, under the control of the *tac* promoter and terminated by the *rrnB* terminator, into the integration locus CgLP11 | - This study |
| P13 | pK19*mobsacBlacI^q^*_*pntAB*_CgLP12_ | Km^r^; pK19*mobsacBlacI^q^* derivate for the integration of *pntAB*, under the control of the *tac* promoter and terminated by the *rrnB* terminator, into the integration locus CgLP12 | This study |
| - P14 | - pEKEx2 | - Km^r^; *E. coli*-*C. glutamicum* shuttle vector (P*_tac_*, *lacI*^q^, pBL1 *oriV_C.g._*, pUC18 *oriV_E.c_*) | - (Eikmanns et al., 1994) |
| - P15 | - pEKEx2-*maqu2220* | - Km^r^; pEKEx2 derivate with coding sequence of *maqu_2220* of *M. hydrocarbonoclasticus* VT8 | - This study |
| - P16 | - pEKEx2-*maqu2507* | - Km^r^; pEKEx2 derivate with coding sequence of *maqu_2507* of *M. hydrocarbonoclasticus* VT8 | - This study |
| - P17 | - pEKEx2-*pntAB* | - Km^r^; pEKEx2 derivate with coding sequence of *pntAB* of *E. coli* K-12 MG1655 | - This study |
| - P18 | - pVWEx1-*xylAB* | - Km^r^; P_tac_, *lacI^q^* pHM1519 *oriV_C.g,_ oriV_E.c._* *C. glutamicum/E. coli* expression shuttle vector for overexpression of *xylA* from *Xanthomonas campestris* SCC1758 and *xylB* from *C. glutamicum* WT | - (Meiswinkel et al., 2013) |

Table S2: Oligonucleotides used in this study. Complementary overlaps are italicized, and synthetic RBS + spacer sequences, as described by Shi et al. (2018), are displayed in bold.

| **No.** | **Primer** | **Purpose** | **Sequence (5’ → 3’)** |
| --- | --- | --- | --- |
| 1 | pK19lacI_fw | Amplification of MCS of pK19*mobsacB*-derived plasmids & sequencing | TCAGTGAGCGAGGAAGCG |
| 2 | pK19lacZ_rv | Amplification of MCS of pK19*mobsacB*-derived plasmids & sequencing | TTAGCAGCCCTTGCGCC |
| 3 | FW_pEKEx2 | Amplification of MCS of pEKEx2-derived plasmids & sequencing | GCACTCCCGTTCTGGATAATGTT |
| 4 | RW_pEKEx2 | Amplification of MCS of pEKEx2-derived plasmids & sequencing | GCGTTCTGATTTAATCTGTATCAGGCTG |
| 5 | FW_upFasR | Amplification of Flank1 of *fasR*; Template: DNA of S3 | *GTCGACTCTAGAGGATCCCC*TGCCTAATGATTCGGGTTCGAC |
| 6 | RV_upFasR | Amplification of Flank1 of *fasR*; Template: DNA of S3 | *CGTGCGTGTTGACTATCCAGTAG*TAAAATTTCAGTGCCTGCCGCT |
| 7 | FW_downFasR | Amplification of Flank2 of *fasR*; Template: DNA of S3 | *AGCGGCAGGCACTGAAATTTTA*CTACTGGATAGTCAACACGCACG |
| 8 | RV_downFasR | Amplification of Flank2 of *fasR*; Template: DNA of S3 | *TGAATTCGAGCTCGGTACCC*GCCGACGGCGCCAACTCTGC |
| 9 | FW_ΔfasR_validation | Validation of *fasR* deletion | CCATATGCAAGGTATCTACATCGAAGAG |
| 10 | RV_ΔfasR_validation | Validation of *fasR* deletion | AACACCCTCCAACAGCTCG |
| 11 | FW_Maqu_2220 | Amplification of *maqu2220*; sequencing | *ATCCCCGGGTACCGAGCTCG***GAAAGGAGAGGATTG**ATGGCAATACAGCAGGTACATCAC |
| 12 | RV_Maqu_2220 | Amplification of *maqu2220*; sequencing | *CTGTAAAACGACGGCCAGTG*TCAGGCAGCTTTTTTGCGC |
| 13 | FW_Maqu_2507 | Amplification of *maqu2507*; sequencing | *ATCCCCGGGTACCGAGCTCG***GAAAGGAGAGGATTG**ATGAATTATTTCCTGACAGGCGGC |
| 14 | RV_Maqu_2507 | Amplification of *maqu2507*; sequencing | *CTGTAAAACGACGGCCAGTG*TTACCAGTATATCCCCCGCATAATCG |
| 15 | FW_TrrnB | sequencing | CTGTTTTGGCGGATGAGAGAAGATT |
| 16 | RV_TrrnB | sequencing | AGGAGAGCGTTCACCGACAA |
| 17 | FW_F1_CgLP11 | Amplification of Flank1 of CgLP11; Template: DNA of S2; sequencing | *GTCGACTCTAGAGGATCCCC*GAATCGGCCTCCGTGGAATTC |
| 18 | RV_F1_CgLP11_universal | Amplification of Flank1 of CgLP11; Template: DNA of S3 | AAATGGGAAAGGCGTGATTCCG |
| 19 | FW_F2_CgLP11_universal | Amplification of Flank2 of CgLP11; Template: DNA of S3; sequencing | CCGCGTTAAAATACCAGGTCAACAC |
| 20 | RV_F2_CgLP11 | Amplification of Flank2 of CgLP11; Template: DNA of S3; sequencing | *TGAATTCGAGCTCGGTACCCC*TGCGATGACATCGATGAGCAATTC |
| 21 | FW_CgLP11_validation | Validation of insertion into CgLP11; sequencing | CGGCTAACGCCGTCTTAAAGG |
| 22 | RV_CgLP11_validation | Validation of insertion into CgLP11; sequencing | CGCCGGTGGAGGTGTC |
| 23 | FW_Ptac_pEKEx_CgLP11 | Amplification of (P*_tac_*-*maqu2220*-T*_rrnB_*); template: P15; sequencing | *CGGAATCACGCCTTTCCCATTT*GAGCTGTTGACAATTAATCATCGGC |
| 24 | RV_Ptac_pEKEx_CgLP11 | Amplification of (P*_tac_*-*maqu2220*-T*_rrnB_*); template: P15 | *GTGTTGACCTGGTATTTTAACGCGG*CAAAAGAGTTTGTAGAAACGCAAAAAGG |
| 25 | FW_lacIq_2 | Amplification of lacI^q^; template: P14 | *ATGGCGAATGGCGCGATAAG*TCAAGCCTTCGTCACTGGTC |
| 26 | RV_laqIq | Amplification of lacI^q^; template: P14 | *GTGCTTGCGGCAGCGTGAAG*GCGGCATGCATTTACGTTGAC |
| 27 | FW_F1_cg2692 | Amplification of Flank1 of cg2692; Template: DNA of S3 | *GTCGACTCTAGAGGATCCCC*CCGCACCTGAGGTGATAGAAAGAG |
| 28 | RV_F1_cg2692GTG | Amplification of Flank1 of cg2692 for GTG start codon mutation; Template: DNA of S3 | TAGGCGAACCCCGTGGCAG |
| 29 | FW_F2_cg2692GTG | Amplification of Flank2 of cg2692 with GTG start codon mutation; Template: DNA of S3 | CTGCCACGGGGTTCGCCTA |
| 30 | RV_F2_cg2692 | Amplification of Flank2 of cg2692; Template: DNA of S3 | *TGAATTCGAGCTCGGTACCC*GATCGCTACAGCTGCGCTCC |
| 31 | RV_F1_cg2692TTG | Amplification of Flank1 of cg2692 for TTG start codon mutation; Template: DNA of S3 | TAGGCGAACCCCTTGGCAGC |
| 32 | FW_F2_cg2692TTG | Amplification of Flank2 of cg2692 with TTG start codon mutation; Template: DNA of S3 | CTGCCAAGGGGTTCGCCTA |
| 33 | FW_cg2692_validation | Validation of start codon mutation of cg2692; sequencing | CGACGCCGTCACGTTGAA |
| 34 | RV_cg2692_validation | Validation of start codon mutation of cg2692; sequencing | GCGGATCCCCATGGTTTAGG |
| 35 | FW_F1_CgLP12 | Amplification of Flank1 of CgLP12; Template: DNA of S3 | *GTCGACTCTAGAGGATCCCC*TGCAGACCGAAGGTGAAATCG |
| 36 | RV_F1_CgLP12_universal | Amplification of Flank1 of CgLP12; Template: DNA of S3 | AATATGCCGATTGCAAGAAACGAGAAG |
| 37 | FW_F2_CgLP12_universal | Amplification of Flank2 of CgLP12; Template: DNA of S3 | CAGTCAAAAAATGTTGAAATCAGCACTTTCA |
| 38 | RV_Flank2_CgLP12 | Amplification of Flank2 of CgLP12; Template: DNA of S3 | *TGAATTCGAGCTCGGTACCCC*AATAATTGGTGCCCCAACTTTTGGA |
| 39 | FW_Ptac_pEKEx2_CgLP12 | Amplification of (P*_tac_*-*pntAB*-T*_rrnB_*); template: P17 | *CTTCTCGTTTCTTGCAATCGGCATATT*GAGCTGTTGACAATTAATCATCGGC |
| 40 | RV_TrrnB_pEKEx2_CgLP12 | Amplification of (P*_tac_*-*pntAB*-T*_rrnB_*); template: P17 | *TGAAAGTGCTGATTTCAACATTTTTTGACTG*CAAAAGAGTTTGTAGAAACGCAAAAAGG |
| 41 | FW_pntAB_Ec_pEKEx2 | Amplification of *pntAB*; template: S2 | *ATCCCCGGGTACCGAGCTCG***GAAAGGAGAGGATTG**ATGCGAATTGGCATACCAAGAGAAC |
| 42 | RV_pntAB_Ec_pEKEx2 | Amplification of *pntAB*; template: S2 | *CTGTAAAACGACGGCCAGTG*TTACAGAGCTTTCAGGATTGCATCCA |
| 43 | FW_pntAB_Ec | Sequencing | *GAAACCTACGAAAGGATTTTTTACCC*ATGCGAATTGGCATACCAAGAGAAC |
| 44 | RV_pntAB_Ec | Sequencing | *AATCTTCTCTCATCCGCCAAAACAG*TTACAGAGCTTTCAGGATTGCATCCA |
| 45 | FW_pntAB_Ec_seq1 | Sequencing | CGCGGAATTCCTCGAGCTG |
| 46 | FW_pntAB_Ec_seq2 | Sequencing | GCTTCCTTAGTTTTATCGCGGTGC |
| 47 | FW_pntAB_Ec_seq3 | Sequencing | GCGGGCTTTATGCTCAGCAA |
| 48 | FW_seq1_F1_CgLP12 | Sequencing | CGCACCAGTCCCATTCCAC |
| 49 | FW_seq1_F2_CgLP12 | Sequencing | TTGGATTTGTGCTTTTACTTGTCTCG |
| 50 | FW_gltA_validation | Validation of *gltA* promotor exchange; sequencing | AGCCAAGGAGCAAGCTTAGAAG |
| 51 | RV_gltA_validation | Validation of *gltA* promotor exchange; sequencing | CATGAGGCGAACGGTTGC |
| 52 | *actA*-UF-fw | Amplification of Flank 1 of *actA*; Template: DNA of S3 | GGTCTCTCAGAACCTAACTATTGTCCCCCGTGAATC |
| 53 | *actA*-UF-rv | Amplification of Flank 1 of *actA*; Template: DNA of S3 | GGTCTCATAGTTGAATACCTCCTCAGGTAATCGGAC |
| 54 | *actA*-DF-fw | Amplification of Flank 2 of *actA*; Template: DNA of S3 | GGTCTCTCATAGTTTTTTCTTGGTTTAGAAACCGCCG |
| 55 | *actA*-DF-rv | Amplification of Flank 2 of *actA*; Template: DNA of S3 | GGTCTCAATACCACCACGATAACGCGGACG |
| 56 | *xylAB*-fw | Amplification of *xylAB*; template: P18 | GGTCTCTACTAATGAGCAACACCGTTTTCATCG |
| 57 | *xylAB*-rv | Amplification of *xylAB*; template: P18 | GGTCTCATATGCTAGTACCAACCCTGCGTTGC |
| 58 | *actA*-UF-g2 | Verification and sequencing of *xylAB* integration | CTCATTGCGCTCAACAAGAGC |
| 59 | *actA*-DF-g2 | Verification and sequencing of *xylAB* integration | GACCGTCGTCTTCGGAAGAG |
| 60 | gX-fw | Amplification of gX module; ; Template: DNA of S11 | *GCTTGCATGCCTGCAGGTCGACTCTAG*AGACCTAACTATTGTCCCCCGTGAATC |
| 61 | gX-rv | Amplification of gX module ; Template: DNA of S11 | *GGCCAGTGAATTCGAGCTCGGTACCCGGG*CACCACGATAACGCGGACGGG |
| 62 | *xylA*-qrt-fw | qRT-PCR fwd primer for analysis of *xylA* expression | GGTGTACGACGCCAACAAG |
| 63 | *xylA*-qrt-rv | qRT-PCR rv primer for analysis of *xylA* expression | GTGAAAGCAGTAATACGGCACG |
| 64 | *xylB*-qrt-fw | qRT-PCR fwd primer for analysis of *xylB* expression | GCGTGGAATGGGAAGAGTTC |
| 65 | *xylB*-qrt-rv | qRT-PCR rv primer for analysis of *xylB* expression | CATCATCCAATGCCAACAACAAG |
| 66 | *sigA*-qrt-fw | qRT-PCR fwd primer for analysis of *sigA* expression | AACCATCCGTATCCCAGTCC |
| 67 | *sigA*-qrt-rv | qRT-PCR rv primer for analysis of *sigA* expression | AGTCTTCGATGAAGTCGCC |

**
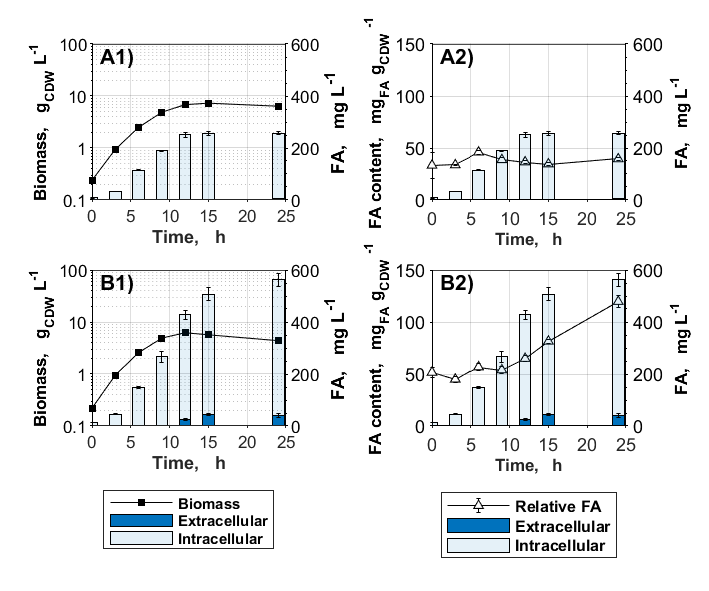
**

Figure S1: Influence of *fasR* deletion on fatty acid production under nitrogen-limiting conditions. Cultivations were conducted in the nitrogen-limiting NL-CgXII medium containing per liter 20 g glucose as the carbon source. A) *C. glutamicum* WT; B) *C. glutamicum* Δ*fasR*. Data represent means of ≥ 3 biological replicates with standard deviations.


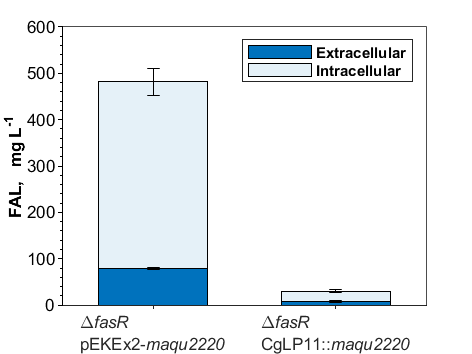


Figure S2: Fatty alcohol production of plasmid-harboring and plasmid-free *C. glutamicum* Δ*fasR* mutants with the FAR Maqu2220. Cultivations were conducted in NL-CgXII medium containing 20 g glucose L^-1^. Samples for FAL analysis were taken after 48 h. Data represent means of 3 biological replicates with standard deviations.


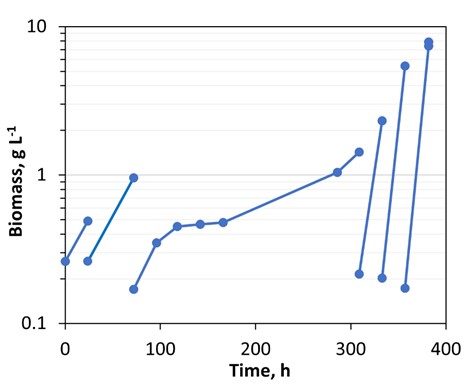


Figure S3: ALE of *C. glutamicum* Δ*actA*::*xylAB* in shaking flasks in 6 serial batch cultivations over 382 h in total, using CgXII medium containing 40 g xylose L^-1^ as sole carbon source. Transfers to fresh CgXII medium were performed when OD_600_ surpassed at least 2 after 24 h or more.


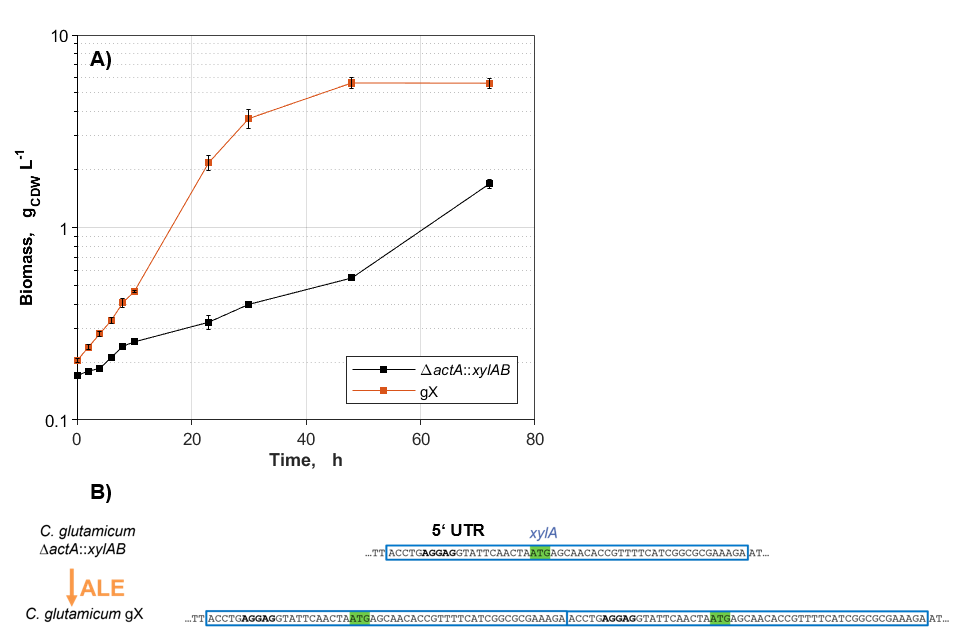


Figure S4: ALE of *C. glutamicum* Δ*actA*::*xylAB* on xylose. A) Growth curves of *C. glutamicum* Δ*actA*::*xylAB* and the evolved strain gX. Cultivations were conducted in CgXII medium containing 40 g xylose L^-1^ as the sole carbon source. Data represent means of 3 biological replicates with standard deviations. B) Nucleotide sequence of the 5’ untranslated regions (UTR) of *C. glutamicum* Δ*actA*::*xylAB* and *C. glutamicum* gX. The duplicated region, comprising 21 bp of the 5’UTR and 32 bp of the beginning of xylose isomerase gene *xylA*, is highlighted (blue box) in the parental strain *C. glutamicum* Δ*actA*::*xylAB* and in the evolved strain gX. The putative RBS (Pfeifer-Sancar et al., 2013) is indicated in bold letters, and the start codon of *xylA* is highlighted in green.


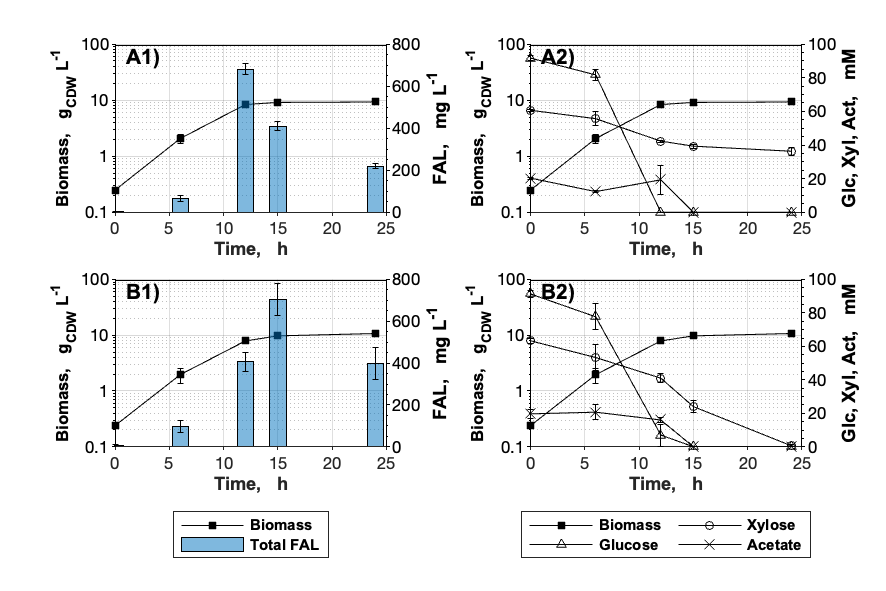


Figure S5: FAL production on wheat straw hydrolysate. Cultivations were conducted in CgXII medium supplemented with 195 µM PCA. The carbon source was provided by hydrolysate, normalized to a concentration of 20 g glucose L^-1^. A) *C. glutamicum* Δ*fasR* cg2692_TTG_ (pEKEx2-*maqu2220*); B) *C. glutamicum* Δ*fasR* cg2692_TTG_ gX (pEKEx2-*maqu2220*). Data represent means of 3 biological replicates with standard deviations.

Abe, S., Takayama, K.-I., & Kinoshita, S. (1967). Taxonomical studies on glutamic acid-producing bacteria. *The Journal of General and Applied Microbiology*, *13*(3), 279-301. <https://doi.org/10.2323/jgam.13.279>

Blattner, F. R., Plunkett, G., Bloch, C. A., Perna, N. T., Burland, V., Riley, M., Collado-Vides, J., Glasner, J. D., Rode, C. K., Mayhew, G. F., Gregor, J., Davis, N. W., Kirkpatrick, H. A., Goeden, M. A., Rose, D. J., Mau, B., & Shao, Y. (1997). The complete genome sequence of *Escherichia coli* K-12. *Science*, *277*(5331), 1453-1462. <https://doi.org/10.1126/science.277.5331.1453>

Eikmanns, B. J., Thum-Schmitz, N., Eggeling, L., Lüdtke, K.-U., & Sahm, H. (1994). Nucleotide sequence, expression and transcriptional analysis of the *Corynebacterium glutamicum gltA* gene encoding citrate synthase. *Microbiology*, *140*(8), 1817-1828. <https://doi.org/10.1099/13500872-140-8-1817>

Hanahan, D. (1983). Studies on transformation of *Escherichia coli* with plasmids. *Journal of Molecular Biology*, *166*(4), 557-580. <https://doi.org/10.1016/S0022-2836(83)80284-8>

Kerbs, A., Mindt, M., Schwardmann, L., & Wendisch, V. F. (2021). Sustainable production of N-methylphenylalanine by reductive methylamination of phenylpyruvate using engineered *Corynebacterium glutamicum*. *Microorganisms*, *9*(4). <https://doi.org/10.3390/microorganisms9040824>

Kinoshita, S., Nakayama, K., & Akita, S. (1958). Taxonomical study of glutamic acid accumulating bacteria, *Micrococcus glutamicus* nov. sp. *Bulletin of the Agricultural Chemical Society of Japan*, *22*(3), 176-185. <https://doi.org/10.1080/03758397.1958.10857463>

Meiswinkel, T. M., Gopinath, V., Lindner, S. N., Nampoothiri, K. M., & Wendisch, V. F. (2013). Accelerated pentose utilization by *Corynebacterium glutamicum* for accelerated production of lysine, glutamate, ornithine and putrescine. *Microbial biotechnology*, *6*(2), 131-140. <https://doi.org/10.1111/1751-7915.12001>

Pérez-García, F., Peters-Wendisch, P., & Wendisch, V. F. (2016). Engineering *Corynebacterium glutamicum* for fast production of l-lysine and l-pipecolic acid. *Applied Microbiology and Biotechnology*, *100*(18), 8075-8090. <https://doi.org/10.1007/s00253-016-7682-6>

Pfeifer-Sancar, K., Mentz, A., Rückert, C., & Kalinowski, J. (2013). Comprehensive analysis of the *Corynebacterium glutamicum* transcriptome using an improved RNAseq technique. *Bmc Genomics*, *14*(1), 888. <https://doi.org/10.1186/1471-2164-14-888>

Schäfer, A., Tauch, A., Jäger, W., Kalinowski, J., Thierbach, G., & Pühler, A. (1994). Small mobilizable multi-purpose cloning vectors derived from the Escherichia coli plasmids pK18 and pK19: selection of defined deletions in the chromosome of Corynebacterium glutamicum. *Gene*, *145*(1), 69-73. <https://doi.org/10.1016/0378-1119(94)90324-7>

Schwardmann, L. S., Dransfeld, A. K., Schäffer, T., & Wendisch, V. F. (2022). Metabolic engineering of *Corynebacterium glutamicum* for sustainable production of the aromatic dicarboxylic acid pipicolinic acid. *Microorganisms*, *10*(4). <https://doi.org/10.3390/microorganisms10040730>

Shi, F., Luan, M., & Li, Y. (2018). Ribosomal binding site sequences and promoters for expressing glutamate decarboxylase and producing γ-aminobutyrate in *Corynebacterium glutamicum*. *AMB Express*, *8*(1), 61. <https://doi.org/10.1186/s13568-018-0595-2>

van Ooyen, J., Noack, S., Bott, M., Reth, A., & Eggeling, L. (2012). Improved L-lysine production with *Corynebacterium glutamicum* and systemic insight into citrate synthase flux and activity. *Biotechnology and Bioengineering*, *109*(8), 2070-2081. <https://doi.org/10.1002/bit.24486>
